# Polar groups, charge density, and backbone flexibility as molecular determinants of CO_2_ capture efficiency and absorption kinetics of polymerized ionic liquids

**DOI:** 10.1039/d6ra05244d

**Published:** 2026-09-28

**Authors:** Ahmed Mohamed Abdelmagid, Abobakr Khidir Ziyada, Abdalla Ahmed Elbashir, Zakaria A. Salih

**Affiliations:** a Department of Chemical Engineering and Chemical Technology, Faculty of Engineering and Technology, University of Gezira Wad Medani 21113 Sudan bahooti@gmail.com; b General Studies Department, Jubail Industrial College Jubail Industrial City 31961 Saudi Arabia; c Department of Chemistry, College of Science, King Faisal University P.O.Box 400 Al-Ahsa 31982 Saudi Arabia aaeahmed@kfu.edu.sa; d Research and Training Station, King Faisal University P.O.Box 400 Al-Ahsa 31982 Saudi Arabia

## Abstract

This study investigates the synthesis, characterization, and CO_2_ capture efficacy of eight structurally varied polymeric ionic liquids (PILs) across four cationic head-group families—ammonium, phosphonium, imidazolium, and pyridinium—each comprised of distinctive functional groups to investigate structure–property correlations in CO_2_ capture. The synthesized PILs are: poly(allyl tris(2-hydroxyethyl)ammonium chloride) [P[AyTHEA][Cl]], poly(4-vinylbenzyl trimethylammonium chloride) [P[4-VBTMA][Cl]], poly(4-vinylbenzyl triethylammonium chloride). [P[4-VBTEA]]Poly[2-(methacryloyloxy)ethyl trimethylammonium chloride] [P[MOETMA][Cl]], poly(4-vinylbenzyl)bis(dimethylamino)phosphino-trimethylammonium chloride [P[4-VBPDMA-TMA][Cl]], poly[(4-methylbenzyl)tris(dimethylamino)phosphonium chloride] [P[4-MBP(DMA)_3_][Cl]], poly(1-allyl-3-propanenitrile imidazolium chloride) [P[AyPrCNIm][Cl]], and poly(allyl pyridinium chloride) [P[AyPyr][Cl]]. Structural verification was achieved using ^1^H NMR spectroscopy and elemental (CHNS) analysis, while water content was determined *via* coulometric Karl Fischer titration. CO_2_ solubility was investigated gravimetrically using magnetic suspension balance (MSB) at temperatures ranging from 298.15 to 358.15 K and at pressures of 1 to 20 bar. The time-dependent uptake was analyzed using a first-order saturation model, resulting in equilibrium mole fractions (*x*_eq_), first-order rate constants (*k*), and half-saturation periods (*t*½) for each PIL. The CO_2_ sorption isotherms exhibited nonlinearity and were characterized by the effective Henry's Law constants (*K*^eff^_H_) which were obtained from the dual-mode sorption (DMS) model, with the Henry dissolving coefficient (*k*_D_) fit to zero for all PILs, hence confirming mostly glassy-state, Langmuir-controlled sorption. The bifunctional PIL P[4-VBPDMA-TMA][Cl] exhibited the maximum CO_2_ uptake (*x*_CO_2__ = 0.6156 at 20 bar, 298.15 K), while P[AyPyr][Cl] had the lowest performance. The calculated enthalpy values for the prepared PILs indicated predominantly physical sorption and advantageous regeneration economics. *K*^eff^_H_ was used to estimate the standard thermodynamic solvation functions—Gibbs free energy (Δ*G*^0^_sol_), enthalpy (Δ*H*^0^_sol_), and entropy (Δ*S*^0^_sol_)—over the whole temperature range. The findings highlight distinct structure–activity relationships linking cation architecture, functional-group identity, and polymer backbone flexibility to CO_2_ capture capacity and kinetics in chloride-based PIL systems, offering rational design principles for next-generation PIL sorbents with enhanced performance for CO_2_ capture applications.

## Abstract 2

This study investigates the structure–property correlations in CO_2_ capture of structurally varied polymeric ionic liquids across ammonium, phosphonium, imidazolium, and pyridinium families—each comprised of distinctive functional groups.

## Graphical abstract

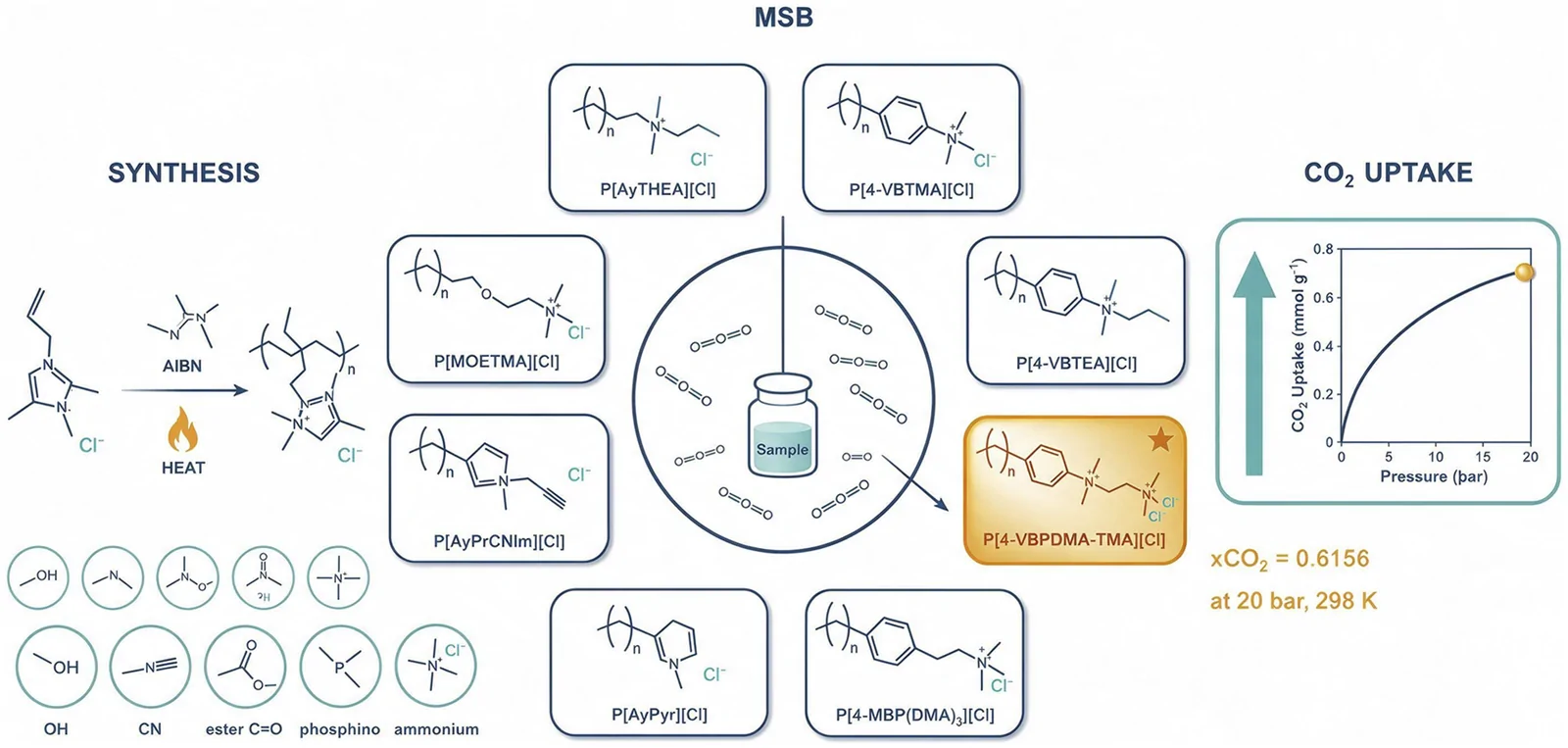

## 1. Introduction

Capturing and lowering the atmospheric CO_2_ content will be crucial over the next few decades.^1–4^ Thus, it is extremely important and necessary from both an economic and environmental perspective to develop new carbon capture materials.^5,6^ Solvents based on aqueous amines are currently widely employed in industrial operations as CO_2_-absorbing solvents. These solvents have a few drawbacks, such as the requirement for energy-intensive heating to regenerate the amine.

Ionic liquids (ILs) have gained immense interest as a promising material for CO_2_ capture in addition to other different engineering applications.^7–9^ ILs are materials that have melting temperatures below 100 °C and typically contain inorganic and/or organic anions and cations^1,10^ with a unique and versatile properties such as excellent solvency for a broad variety of polar and nonpolar molecules, great chemical and thermal stability, minimal vapor pressure, and a broad electrochemical window. These characteristics make ILs promising materials for a wide range of technological and industrial applications, including solar cells, lubricants, electrolytes, biomass processing, electroplating, and CO_2_ capture.^1,11,12^ Some of the main characteristics that make ILs preferable to be used for CO_2_ capture; are their low vapor pressure, low volatility, and high stability when compared with conventional solvents that are already in use.^9,13^ Since ILs can be regenerated at 1 bar, it presents a potentially cost-effective substitute for pre-combustion CO_2_ absorption. In addition, this would minimize the difference in heat transmission between processes and minimize the associated utility costs.^13,14^ Previous studies comparing the performance and cost of the ILs process show that this technology is promising. In addition, having the ability to customize ILs may be used for producing low-cost, thermally stable ILs with excellent CO_2_ capture efficiency and selectivity.^13^ In addition, the elevated temperatures at which ILs degrade protect the equipment from corrosion that may result from ILs if they react with external substances.^1,15–20^ Thus, for the future feasible, affordable, and sustainable CO_2_ capture by ILs, experimental techniques, and data development are crucial.

Polymerized ionic liquids (PILs) exhibit higher CO_2_ absorption than monomeric ILs, making them a potential material for CO_2_ capture.^9,21,22^ Polymerized/polymeric ionic liquids, also known as poly(ionic liquid)s (PILs), are a special type of polyelectrolyte in which each repeating unit includes an IL species, with cationic or anionic centers contained throughout the polymer chain.^21,22^ This structural restriction separates PILs from ordinary polyelectrolytes, in which charges are often delocalized or unevenly dispersed, as well as physical IL-polymer blends. PILs' cation–anion composition may be accurately modified, optimizing CO_2_ solubility, selectivity, and regeneration thermodynamics by incorporating suitable ion-pair chemistry.^21,23–25^ Furthermore, PILs have numerous significant practical advantages over monomeric ILs, including increased mechanical stability, enhanced processability, long-term durability, and spatial controllability of ionic site distribution.^21,26,27^ With a few noted exceptions, PILs are generally solid at normal temperature, while most monomeric ILs are liquids at or near ambient temperature.^28^ The solid-state character at operating conditions is particularly advantageous in gas capture applications since it eliminates the mass-transfer limitations, foaming, and entrainment losses associated with liquid-phase sorbents in packed-column contactors, while also allowing fabrication into membranes, coatings, porous monoliths, and composite adsorbent beds.^29,30^

PILs' improved CO_2_ capture performance is primarily due to the ionic interactions within the polymeric backbone, which increase gas-sorbent affinity beyond what isolated IL ion pairs can achieve, as well as reduced volatility and improved handling characteristics when compared to their monomeric counterparts.^28,31^ Furthermore, the enormous interest in PILs across materials science is due to their capacity to generate novel functionalities by integrating the distinctive properties of ILs—including negligible vapor pressure, wide electrochemical windows, high ionic conductivity, and exceptional thermal and chemical stability—with the structural advantages obtained by macromolecular architecture, such as film-forming ability, mechanical durability, and the prospect of producing structural porosity.^21,32,33^

The current study designed PILs structures to assess the influence of various structural variations on CO_2_ capture efficiency and absorption kinetics. The designed structures comprise characteristics that are known increases CO_2_ capture efficiency in PILs. The cations of the prepared PILs include ammonium, phosphonium, imidazolium, and pyridinium incorporating different types of groups such as phosphino, dimethylamino, trimethylammonium, hydroxyl, nitrile group, methacrylate, ester, and carbonyl groups.

To the best of our knowledge, no study has systematically compared cationic structural diversity across four head-group families—ammonium, phosphonium, imidazolium, and pyridinium—under the controlled condition of a fixed, identical anion, despite the fact that prior studies have examined either anion-effect series (using multiple anions with a fixed cation) or single-family cation series (varying alkyl-chain length within one head-group class). Previous comparative research on multi-PILs have mostly utilized fluorinated anions since these anions provide elevated CO_2_ solubility and reduced viscosity. Nonetheless, this approach hides the impacts of cations, as the anion predominates the isotherm, rendering cation-structural variations seemingly insignificant. The present work isolates cationic structural contributions, including head-group identity, pendant functional-group polarity, cationic charge density, backbone flexibility, and bifunctional site cooperation, as the sole variables governing CO_2_ capture by restricting the anion to Cl^−^ throughout. Moreover, within the ammonium family, the study extends beyond simple alkyl-ammonium PILs to include: (i) a hydroxyl-functionalised variant [P[AyTHEA][Cl]] bearing three –OH groups per repeat unit; (ii) a bifunctional phosphino-ammonium hybrid [P[4-VBPDMA-TMA][Cl]] with dual Lewis basic centers; (iii) a methacrylate-backbone flexible analog [P[MOETMA][Cl]]; and (iv) a nitrile-appended imidazolium [P[AyPrCNIm][Cl]]. The synthesized PILs are distinguished by their structural identity, which is determined by the combination of family variety and within-family functional-group variation inside a fixed-anion series structure. A single internally consistent experimental platform is used to acquire the full isotherm dataset (5 temperatures × 5 pressures × 8 PILs) using MSB gravimetry. This method enables the simultaneous extraction of equilibrium capacities, first-order absorption kinetics, dual-mode sorption parameters, effective Henry's Law constants, and standard solvation thermodynamics. The experimentally substantiated design principles that link molecular architecture directly to both CO_2_ affinity and absorption rate are presented for the first time in a Cl^−^-PIL series by the resulting structure–property relationships.

## 2. Experimental section

### 2.1 Materials

The studied PILs were prepared using Chemicals of analytical grade from Sigma-Aldrich. The CAS number and purity of the chemicals used are as follows: *N*,*N*,*N*-trimethyl-1-(4-vinylphenyl)methanaminium chloride (26616-35-3, 99%), methanol (67-56-1, 99.8%), *N*,*N*-dimethylformamide (DMF) (68-12-2, 99.8%), acetonitrile (75-05-8, 99.8%), triethylamine (121-44-8, 99.5%), sodium salt methyl sulfate (512-42-5, >99.8%), sodium acetate (127-09-3, > 99.8%), magnesium sulfate (7487-88-9, 99.5%), diethyl ether (60-29-7, 99%), 2-trimethylammoniumethyl methacrylate chloride, (5039-78-1, 75 wt% in H_2_O), sodium nitrate (7631-99-4, 99%), 2,6-di-*tert*-butyl-4-methylphenol (DTBMP) (128-37-0, 99%), α,α′-azoisobutyronitrile (AIBN) (78-67-1, 98%), 4-vinylbenzyl chloride (1592-20-7, 90%), tris(dimethylamino)phosphine (1608-26-0, 97%), tetrahydrofuran (THF) (109-99-9, 99.9%), boron trifluoride diethyl etherate (BF_3_OEt_2_)(109-63-7, 98–99%), and dichloromethane (DCM)(75-09-2, 99.8%). All the chemicals were used without additional purification.

### 2.2 Synthesis of polymerized ionic liquids (PILs)

PILs were synthesized using methods described by Tang *et al.*^34,35^Fig. 1 displays the structures for the synthesized PILs. All the PILs shown below were synthesized using a setup consisting of a dry nitrogen purge into a round-bottom flask equipped with a heated oil bath, a nitrogen inlet adapter, a magnetic stirrer, and a reflux condenser, unless otherwise stated.

**Fig. 1:**
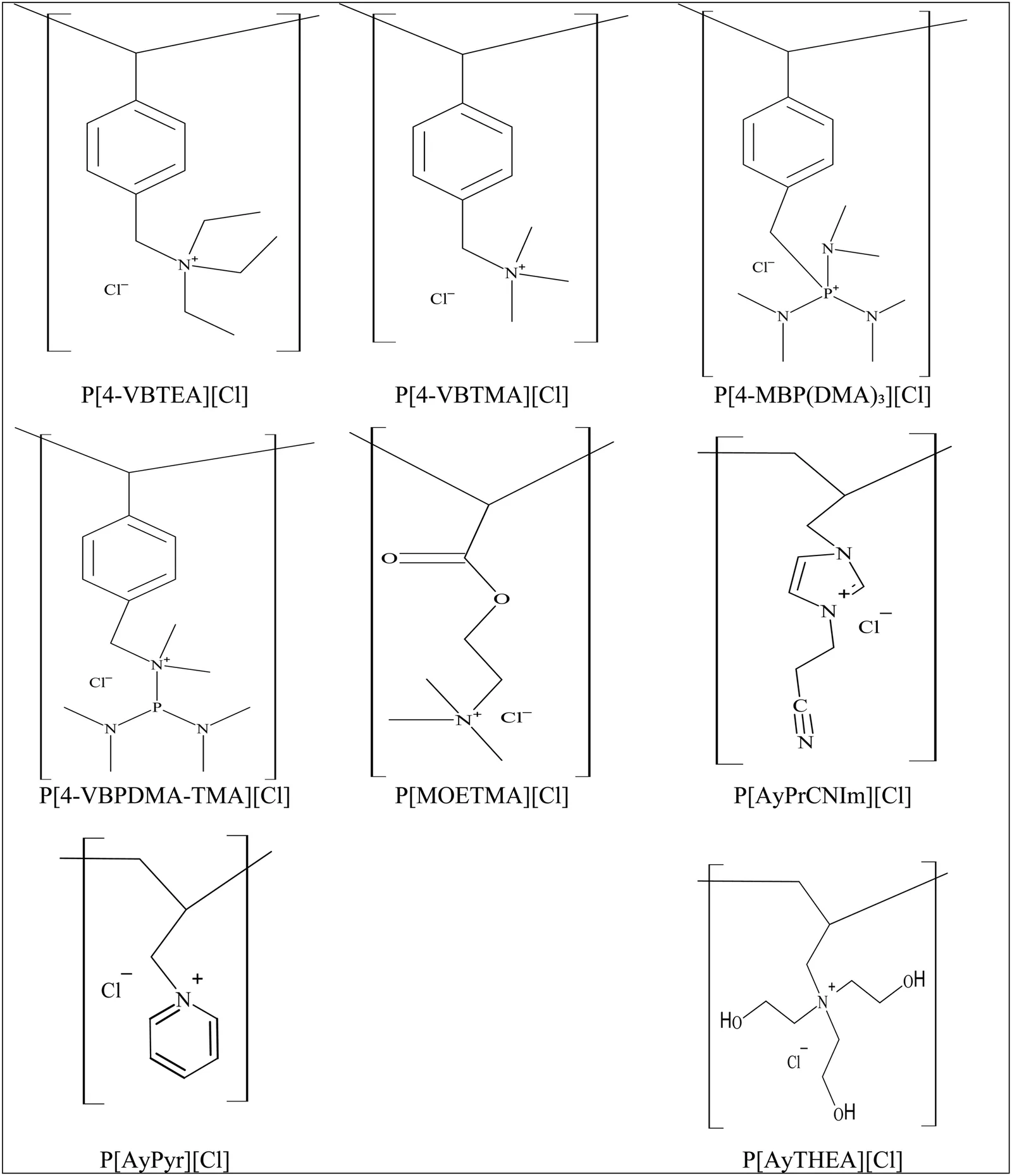
Structures of the synthesized PILs.

The upcoming abbreviations were used for the synthesized PILs; P[4-VBTEA][Cl] for poly(4-vinylbenzyl triethylammonium chloride), P[4-VBTMA][Cl] for poly(4-vinylbenzyl trimethylammonium chloride), P[MOETMA][Cl] for poly[2-(methacryloyloxy)ethyl trimethylammonium chloride], P[4-VBPDMA-TMA][Cl] for poly(4-vinylbenzyl) bis(dimethylamino)phosphino-trimethylammonium chloride, P[4-MBP(DMA)_3_][Cl] for poly[(4-methylbenzyl)tris(dimethylamino)phosphonium chloride], P[AyTHEA][Cl] for poly(allyl tris(2-hydroxyethyl)ammonium chloride), P[AyPrCNIm][Cl] for poly(1-allyl-3-propanenitrile imidazolium chloride), and P[AyPyr][Cl] for poly(allyl pyridinium chloride).

#### 2.2.1 Synthesis of poly ammonium-based ILs

##### 2.2.1.1 Synthesis of poly(allyl tris(2-hydroxyethyl)ammonium chloride (P[AyTHEA][Cl])

0.20 moles of tris-(2-hydroxyethyl) amine were injected to the round-bottom flask, followed by 0.2 moles of allyl chloride and 21 mL of toluene. For 48 hours, the reaction mixture was maintained at 45 °C. The product was cooled to room temperature and rinsed with ethanol. Subsequently, the remaining solvent was removed under vacuum at 80 °C. The product was dried in a vacuum oven for 72 hours to produce allyl tris(2-hydroxyethyl)ammonium chloride ([AyTHEA][Cl]).

0.505 g of Azobisisobutyronitrile “AIBN” was added to [AyTHEA][Cl] in the round-bottom flask, then all were dissolved in 10 mL of dimethylformamide (DMF). The mixture was heated up to 60 °C for 48 hours, then cooled to room temperature. A vacuum oven heated at 100 °C was used to remove the solvent to produce a glassy solid compound (poly(allyl tris(2-hydroxyethyl)ammonium chloride (P[AyTHEA][Cl])).

##### 2.2.1.2 Synthesis of poly trialkyl ammonium-based ILs

4-vinylbenzyl triethylammonium chloride ([4-VBTEA][Cl]) was synthesized in the round-bottomed flask charged with 0.2 mole of 4-vinylbenzyl chloride dissolved in acetonitrile, 0.21 mole of triethylamine, and 0.5 gm of DTBMP. The reaction mixture was heated at 70 °C under a nitrogen atmosphere for 36 h. The product was cooled to room temperature, washed with diethyl ether, and the remaining solvent was removed at 75 °C in a vacuum to afford [4-VBTEA][Cl]. The product was mixed with 60 mg of AIBN and 20 ml of DMF, stirred, and heated at 70 °C under nitrogen atmosphere for 12 h. The solid compound that resulted was dried in a vacuum oven at 100 °C for 48 hours to obtain a glassy solid compound -poly(4-vinylbenzyl triethylammonium chloride (P[4-VBTEA][Cl]).

A similar procedure used to produce P[4-VBTEA][Cl]) was repeated to synthesize the glassy solid compound (poly(4-vinylbenzyl trimethylammonium chloride (P[4-VBTMA][Cl])) by using trimethylamine instead of triethylamine.

##### 2.2.1.3 Synthesis of poly 2-trimethylammoniumethyl methacrylate chloride

Prior to the synthesis of poly[2-(methacryloyloxy)ethyl trimethylammonium chloride] (P[MOETMA][Cl]), water was removed from 2-trimethylammoniumethyl methacrylate chloride under vacuum. The polymerization process was carried out using the same procedure adopted to obtain the glassy solid compound P[4-VBTEA][Cl] to afford P[MOETMA][Cl].

##### 2.2.1.4 Synthesis of poly (4-vinylbenzyl)bis(dimethylamino)phosphinous amide trimethylammonium chloride

4-vinylbenzyl-bis(dimethylamino)phosphino-trimethylammonium chloride ([4-VBPDMA-TMA][Cl]) was synthesized in a round-bottomed flask flushed with dry nitrogen and equipped with a cooling ice bath, reflux condenser, nitrogen inlet adapter, and magnetic stirrer. The flask was charged with 0.2 mole of tris(dimethylamino)phosphine and BF_3_OEt_2_ (added dropwise) dissolved in THF. The mixture was stirred while maintaing the temperature at 0 °C for 1 hour. To the flask, MeOH was added followed by 4-vinylbenzyl chloride dissolved in MeOH and 0.5 gm of DTBMP. The ice bath was removed and the reaction continuously stirred at 65 °C for 36 h. The product was cooled to room temperature, washed with sodium hydroxide, then the product was extracted using DCM and dried over anhydrous magnesium sulfate. A similar procedure used to synthesize P[4-VBTEA][Cl] was followed to prepare the glassy solid compound poly(4-vinylbenzyl) bis(dimethylamino)phosphino-trimethylammonium chloride (P[4-VBPDMA-TMA][Cl]) by replacing [4-VBTEA][Cl] with [4-VBPDMA-TMA][Cl].

#### 2.2.2 Synthesis of poly(1-allyl-3-propanenitrile imidazolium chloride) (P[AyPrCNIm][Cl])

The round bottom flask was charged with imidazole (0.5 mol) in methanol (1.0 mol) and mixed with acrylonitrile (0.6 mol). The solution was heated at 50–55 °C for 10 hours and then cooled to room temperature. The volatile materials were removed under vacuum at 70 °C. The viscous liquid left was propanenitrile imidazole. Nitrile functionalized IL was synthesized by direct quaternization reaction of the propanenitrile imidazole with allyl chloride. Allyl chloride (0.5 mol) was added to the propanenitrile imidazole. The resulting compound was cooled to room temperature and washed with ethyl acetate three times and the remaining solvent was removed at 80 °C in a vacuum. It was then dried in a vacuum oven for 72 hours to produce 1-propanenitrile 3-allyl-imidazolium chloride.^36^ For polymerization, the round-bottom flask was loaded with 1-propanenitrile 3-allyl-imidazolium chloride (30 mL), AIBN (0.6 g). All were dissolved in 8 mL of dimethylformamide (DMF), and the mixture was stirred under nitrogen atmosphere at 60 °C for 48 hours, and then cooled to room temperature. The volatile materials were removed under vacuum at 100 °C to obtain a glassy solid compound P[AyPrCNIm][Cl]).

#### 2.2.3 Synthesis of poly(allyl pyridinium chloride) (P[AyPyr][Cl])

The synthesis of the IL comprised 0.20 mol of allyl chloride and 0.19 mol of pyridine, both dissolved in 20 mL of ethyl acetate. The solution was subsequently stirred and heated to 40 °C in the round-bottom flask. The product was subjected to vacuum drying at 80 °C to remove the solvent, yielding allyl pyridinium chloride [AyPyr][Cl]). To the round bottom flask, 43.93 mL of [AyPyr][Cl], 0.44 g AIBN, and 19.4 ml of dimethylformamide (DMF) were added and stirred in a nitrogen flow for 48 hours at 60 °C. The mixture was cooled to room temperature, then the solvent was evaporated in vacuum at 100 °C to produce a glassy solid compound P[AyPyr][Cl]).

#### 2.2.4 Synthesis of poly[(4-methylbenzyl)tris(dimethylamino)phosphonium chloride] (P[4-MBP(DMA)_3_][Cl])

4-methylbenzyl)tris(dimethylamino)phosphonium chloride ([4-MBP(DMA)_3_][Cl]) was synthesized in a round-bottomed flask equipped with a cooling ice bath. The flask was charged with 0.2 mole of tris(dimethylamino)phosphine dissolved in THF and 0.5 gm of DTBMP, the solution was allowed to cool to 0 °C under stirring. 0.2 mole of 4-vinylbenzyl chloride was added dropwise maintaing the addition rate below 0.2 mL min^−1^. After complete addition of the 4-vinylbenzyl chloride, the ice bath was removed and the reaction continuously stirred at room temperature for 36 h. The product was cooled to room temperature, washed with diethyl ether, and the remaining solvent was removed in vacuum to afford [4-MBP(DMA)_3_][Cl]. A similar procedure used to synthesize P[4-VBTEA][Cl] was followed to prepare a glassy solid compound (P[4-MBP(DMA)_3_][Cl] by replacing [4-VBTEA][Cl] with [4-MBP(DMA)_3_][Cl].

### 2.3 Characterization

#### 2.3.1 NMR and elemental analysis

The ^1^H NMR spectra of the synthesized ionic liquids (ILs) were obtained in dimethyl sulfoxide (DMSO) as the solvent, utilizing Agilent 600 MHz Premium COMPACT nuclear magnetic resonance NMR Magnet spectrophotometer. The spectra were obtained at ambient temperature, using suitable pulse sequences and relaxation delays to guarantee comprehensive signal averaging and optimal resolution; chemical shifts were internally referenced to the residual proton signal of DMSO (*δ* = 2.50 ppm). The resultant spectra were processed and analyzed with conventional NMR software to assign proton environments and verify the structure of the synthesized PILs.

The elemental analysis (CHNS) of the synthesized PILs was performed using a CHNS-932 elemental analyzer from LECO Instruments. The device was calibrated before each measurement series with a recognized standard reference material of known chemical composition provided by the manufacturer, ensuring precise quantification of carbon, hydrogen, nitrogen, and sulfur levels. The sample was accurately weighed inside a tin or ceramic capsule and placed into the analyzer, where it was combusted at elevated temperatures in an oxygen flow. The resultant gases (CO_2_, H_2_O, N_2_, and SO_2_, when applicable) were isolated and analyzed to determine the percentage content of each constituent. The experimental results were subsequently compared with the theoretical values derived from the designated molecular formula.

#### 2.3.2 Water contents

The water content of the synthesized PILs was measured by coulometric Karl Fischer titration, conducted with a DL 39 Coulometric Titrator (Mettler Toledo). The titration utilized a combination of CombiCoulomat fritless Karl Fischer reagent (Merck) as the anolyte/electrolyte system and Hydranal Coulomat AG reagent (Riedel-de Haën) as the catholyte, ensuring heightened sensitivity and reproducibility for trace moisture analysis. Before each measurement, the device was calibrated and the background drift was stabilized to reduce instrumental error. All samples were accurately weighed into the titration cell, and triplicate independent measurements were performed for each PIL. The resultant water contents were averaged throughout the three trials and reported to indicate the moisture level and overall purity of the PILs.^37^

#### 2.3.3 Thermal analysis and morphology

Thermal properties of the prepared PILs were studied by Simultaneous Thermal Analyzer (STA) 6000 with a heating rate (5 °C min^−1^). The STA 6000 Simultaneous Thermal Analyzer measures and analyzes sample weight change and heat transfer in real time. Comparative thermal analysis (DTA), alternatively referred to as differential scanning calorimetry (DSC), and thermogravimetric analysis (TGA) can be performed concurrently with this instrument. The temperature, heat flow and weight calibrations were verified using the samples of indium and silver (which were provided in the Spares and Accessory Kit with the instrument) before obtaining data. The following order was followed when performing the calibration; Weight Calibration and Verification, Sensor Calibration, DTA Baseline Optimization, Temperature Verification and Heat Flow Verification.

Scanning electron microscopy (SEM) is the most used technique for microstructural investigation nowadays. This approach focuses electrons accelerated by high voltage (0–30 kV) on the material. Electrons and material atoms interact when the concentrated electron beam scans its surface. Detectors gather the electrons and X-rays produced by this collision. The detector converts these impulses into digital signals, which are shown on the computer screen. The image displayed on the screen provides information about the microstructure of the sample. Scanning electron microscopy (SEM) was conducted using a Thermo Scientific Prisma scanning electron microscope (SEM). The apparatus is equipped with a tungsten electron source and conventional detectors, comprising an Everhart–Thornley secondary-electron detector (ETD), a low-vacuum secondary-electron detector (LVD), a gaseous secondary-electron detector (GSED) for ESEM functionality, and an infrared (IR) camera for observing samples within the chamber. Imaging was performed in high-vacuum, low-vacuum, or SEM mode, contingent upon the conductivity and preparation of the sample.

### 2.4 CO_2_ solubility in poly ILs

The experimental solubility of CO_2_ in the produced PILs was measured using the gravimetric technique with a magnetic suspension balance (MSB). This technique is often used to evaluate the solubility of gases in solids (by measuring the change in mass of the sample after gas uptake), but is seldom used for liquids since solvent evaporation can heavily affect the measured mass and lead to enormous inaccuracies. However, evaporation effects were limited, such that the gravimetric technique could be used to determine the solubility of CO_2_ in these non-volatile solvents, due to the very low volatility and vapor pressure of the ILs under the experimental conditions.^38^ This technique enables a direct mass-based measurement of the absorbed CO_2_ and offers a clear thermodynamic basis for solubility data.

The magnetic suspension balance (MSB) is a flexible and extremely accurate tool for investigating the solubility of gases in PILs. It allows continuous monitoring of mass changes in real time with a high time-based precision, which is required in order to detect the start of equilibrium and to ensure that the absorption process has achieved a stable state. True equilibrium must be achieved as solubility values calculated from non-equilibrium data may be significantly over or under-estimated. The MSB may also be used to verify that the original sample is fully degassed by measuring that no additional change in mass takes place after extended exposure to vacuum or inert gas purge, another need for accurate and repeatable solubility measurements.

The CO_2_ solubility in the prepared PILs was measured with a magnetic suspension balance (MSB) from Rubotherm Präzisionsmesstechnik GmbH (Bochum, Germany). The instrument employs a magnetic suspension coupling system composed of an electromagnet and a suspension magnet. The electromagnet is electronically controlled to maintain the suspension magnet in a frictionless levitation state while transmitting the sample mass without contact. The microbalance was said to be precise to 0.001 mg and reproducible to ± 0.020 mg. This permitted very accurate monitoring of minor mass changes during gas absorption. The pressure and temperature in the high-pressure measuring cell were regulated and maintained within the limits of ±0.05 bar and ±0.2 °C, respectively,^37^ so that the circumstances in each measurement nearly matched the target thermodynamic state points. All possible environmental disturbances that could affect the sample mass were minimized, controlled, and quantified (*e.g.* temperature changes, external vibrations, and air current effects) in order to obtain highly accurate weight measurements. The buoyancy effects that are also a function of pressure and temperature were explicitly calculated and corrected using Rubotherm's MessPro software that controls the instrument, acquires the data, and performs the real-time buoyancy compensation.

The MSB method for the determination of CO_2_ solubility in the synthesized PILs was carried out in four phases including blank measurement, sample drying, buoyancy calibration and solubility measurement. The blank measurement consists of *in situ* weighing of the empty sample container (usually a high pressure-compatible steel or sapphire cell) under vacuum or inert atmosphere, and measuring its effective volume either by geometric calibration or by a separate buoyancy measurement in a reference fluid. The PIL sample is then added to the container and dried under vacuum at increased temperature (if needed) to eliminate residual water and volatile contaminants that may interfere with the mass-based solubility assessment. The volume of the liquid sample contained in the vessel is then accurately measured using a buoyancy based volumetric measurement, which is needed to translate the change in mass to a molar or mass based solubility value. The measurement of solubility itself starts at a constant temperature, to which the pressure is slowly increased to the target amount in regulated increments, continually monitoring the system. The mass of the IL sample is recorded over time until the MSB no longer increases in mass within a given time period, indicating that CO_2_ absorption has reached equilibrium. The mass increase is then used to determine the equilibrium solubility of CO_2_ in the IL at the specified pressure and temperature, with buoyancy adjusted values automatically provided by the MessPro program. The whole experimental setup, including the design of the high-pressure cell, the sample loading procedure, the temperature and pressure management, and the data gathering technique, is detailed in depth in the linked literature.^37,39,40^ Moreover, more detailed MSB experimental procedure of the measurement is reported in the SI under the title CO_2_ solubility measurement using MSB.

## 3. Results

### 3.1 NMR and elemental analysis

The structures of the produced PILs were verified using ^1^H NMR spectra and elemental analysis. The findings were consistent with the anticipated molecular structures, confirming the effective synthesis of the target PILs and supporting the assigned chemical compositions. The ^1^H NMR and elemental analytical findings for the prepared PILs (P[4-VBTEA][Cl], P[4-VBTMA][Cl], P[4-MBP(DMA)_3_][Cl], P[4-VBPDMA-TMA][Cl], P[MOETMA][Cl], P[AyPrCNIm][Cl], P[AyTHEA][Cl], and P[AyPyr][Cl]) are provided in the SI (Fig. S1–S8).

The ^1^H NMR spectra provide corroborative evidence for the synthesis of the PILs by the polymerization of allylic, vinylic, or methacrylate moieties. Specifically, polymerization is anticipated to use the monomeric C=C bonds, leading to the vanishing or significant reduction of the associated alkene proton resonances in the about 5.0–6.5 ppm range. This alteration ought to be followed by the emergence of extensive aliphatic resonances attributed to the newly synthesized saturated polymer backbone. The resonances linked to aromatic rings, ammonium or phosphonium moieties, imidazolium or pyridinium structures, hydroxyl functionalities, nitrile groups, and ester groups persist as these entities do not participate directly in the radical propagation process. The widening of the polymer signals aligns with the limited molecular mobility and variation of chemical surroundings typical of polymer chains. However, signal broadening by itself is inadequate to demonstrate the production of polymers. Complementary characterisation using elemental analysis, DSC, TGA, and SEM assessments was conducted to substantiate the claim of polymerization.

### 3.2 Thermal analysis and morphology

A DSC glass transition step-wise change in the baseline, indicative of a variation in heat capacity. It illustrates the transformation of the amorphous polymer phase from a solidified glassy condition to a less rigid, dynamic rubbery condition when subjected to heat. The DSC thermograms of the synthesized PILs are presented in Fig. S9 in the SI. All the prepared PILs exhibited baseline alterations associated with glass transition, without clear melting or crystallization peaks. This conduct implies that the substances are mostly amorphous and that the polymer chains persist in a glassy condition. PILs have a lower crystallization ability, which is caused by the presence of bulky ionic groups, strong electrostatic interactions, and uneven packing of the polymer chains. The lack of a melting endotherm is consistent with this reduced crystallization ability. The findings from the DSC provide support to the claim that amorphous polymeric ionic compounds are formed. Comparable PILs investigations have shown a glass-transition signal without a melting peak, leading to the classification of the materials as primarily possessing an amorphous polymeric structure.^1^

Thermogravimetric analysis (TGA) was conducted on the synthesized PILs in order to assess their polymeric features. The TGA graphs of the synthesized PILs are presented in Fig. S10 in the SI all samples demonstrated a uniform breakdown pattern characterized by an initial stable plateau from 50 to about 200 °C, during which no mass loss occurred apart from a slight contribution from adsorbed moisture, affirming the thermal resilience of the materials far above ambient temperatures. This plateau was succeeded by two distinct decomposition phases: an initial moderate mass-loss occurred between approximately 200 and 320 °C (residual mass 55–70%), followed by a significantly larger mass-loss between roughly 380 and 470 °C, after which a stable char plateau remained until 700 °C, preserving between 10% and 40% of the original sample mass. This dual-phase decomposition characteristic, along with a significant and thermally stable char residue, is commonly acknowledged as indicative of polymeric substances containing pendant quaternary ammonium groups, contrasting with the thermal behavior anticipated from a discrete small-molecule ionic liquid, which generally experiences a singular, sharp decomposition or volatilization event with minimal to no remaining mass at elevated temperatures. The mechanistic categorization of the two phases is well-documented in the literature concerning poly(vinylbenzyl ammonium)-type polyelectrolytes.^1^ The combined two-step decomposition process—including the removal of distinct ionic side groups followed by the decomposition of the backbone—together with the significant char residue at elevated temperatures, provide strong thermogravimetric proof of the polymeric characteristics of these substances.

The SEM pictures of the synthesized PILs are presented in Fig. S11 in the SI. The SEM analysis of the prepared PILs revealed that the PIL samples had continuous, variable surface morphologies with sample-dependent roughness and apparent void structure. The micrographs appear to show continuous, irregular, and non-crystalline-looking surfaces with variations in roughness and apparent void structure among the PILs. Such differences are reasonable because the anion, ionic-group substituent, backbone structure, and degree of crosslinking can influence phase separation, chain packing, shrinkage during drying, and the formation of voids. The observed morphologies are compatible with amorphous polymeric ionic materials.

The aforementioned characterization comprises the absence of clear melting transitions in the DSC thermograms, pointing to an amorphous glassy state, along with the brittle, conchoidal fracture surfaces observed by SEM, which is characteristic of glassy polymeric solids. Taken together, these two independent lines of evidence support the conclusion that the materials under study are amorphous, glassy PILs rather than small-molecule ionic liquids.

### 3.3 Solubility of CO_2_ in PILs

The study aimed to compare the CO_2_ capture affinity of PILs with distinct cationic head groups incorporating similar anion. Cations play a significant role in determining PIL characteristics, while anions are more decisive in the case of ILs.^32^ This allows for a clear assessment of cation structural effects on CO_2_ capture efficiency.

This study examines the cationic diversity of four chemically distinct head group families: ammonium, pyridinium, phosphonium, and imidazolium. Each family differs in charge delocalization, electronic density distribution, steric profile, and ability to interact with CO_2_ molecules specifically. These cationic head groups were further functionalized with different characteristic functional groups such as allyl, vinylbenzyl, nitrile, and carboxylate, chosen for their contrasting electronic properties and expected influence on the polarity, hydrogen-bonding capacity, and gas–solvent interaction strength of the resulting PIL. The PILs studied are P[AyTHEA][Cl], P[4-VBTMA][Cl], P[4-VBTEA][Cl], P[4-MBP(DMA)_3_][Cl], P[4-VBPDMA-TMA][Cl], P[MOETMA][Cl],P[AyPyr][Cl], and P[AyPrCNIm][Cl], where the abbreviations represent the nature of the functional group and the cationic platform used. The structural variation across cation types and functionality establishes a multidimensional structure–property relationship. This provides mechanistic insight into how the molecular structure of the cationic component governs CO_2_ uptake in the studied PILs systems.

To assess the CO_2_ capture efficacy of each PIL, experimental gas solubility measurements were performed throughout a broad spectrum of thermodynamic conditions. Measurements were conducted at five specific temperatures—298.15, 313.15, 328.15, 343.15, and 358.15 K—covering a range that includes both near-ambient and somewhat higher thermal conditions typical of post-combustion flue gas streams and process operating settings. Solubility data were obtained at five specific CO_2_ pressures—1, 5, 10, 15, and 20 bar—at each temperature, providing a pressure profile that illustrates Henry's Law behavior at low pressures. Tables S1 through S8 in the (SI) detailed the time development of CO_2_ uptake, which reflects the kinetic absorption behavior of each PIL under the applied conditions, in addition to equilibrium solubility values.

#### 3.3.1 Impact of pressure and time on the CO_2_ capture

Fig. 2 displays the solubility profiles of the synthesized PILs, showing the CO_2_ mole fraction (*x*_CO_2__) as a function of time. This provides a visual representation of the rate of CO_2_ absorption and the equilibrium plateau reached under the experimental conditions. The experimental solubility results shown in Fig. 2 exhibit a consistent and gradual significant positive correlation between CO_2_ solubility and applied pressure across the investigated PILs. Increasing pressure drives the equilibrium rightward, the system adjusts by increasing the quantity of captured CO_2_ to reach that of the gas phase, precisely as predicted by Le Chatelier's principle.^41^ This is why *x*_eq_ rises with pressure for all eight PILs in the dataset. At the molecular level, increased pressure enhances the frequency and energy of CO_2_ molecular collisions at the gas–liquid interface, thereby extending the quantity of CO_2_ molecules transitioning into the PIL phase per unit time until a new thermodynamic equilibrium is attained at a higher dissolved concentration.^42^ This behavior aligns with Henry's Law, which predicted a linear correlation between gas partial pressure and solubility in dilute solutions, and is reported for CO_2_ dissolution in ILs systems across several structural groups.^43^ In addition to this pressure dependency, the factors influencing CO_2_ solubility in PILs have shown to be significantly more complex than previously indicated by early structure–property models.^44,45^ Initial interpretive frameworks primarily ascribed CO_2_ absorption capacity to the strength of Lewis acid–base interactions between CO_2_ and the IL anion, increased by contributions from free volume within the IL structure;^43^ however, recent experimental and computational evidence has demonstrated that this binary perspective is an oversimplification.^20,46^ Klahn and Seduraman^47^ conclusively shown that neither specific ion–CO_2_ interactions nor free volume considerations alone adequately explain the observed CO_2_ sorption behavior across structurally varied PILs. Instead, they suggest a more intricate and complicated interaction of intermolecular forces, potentially including quadrupole–dipole interactions,^48^ ion-pairing dynamics,^49^ structural reorganization of the PIL network upon gas dissolution,^50^ and entropic contributions,^51^ in determining the overall CO_2_ uptake capacity. These discoveries highlight the need to advance from single-parameter models to a more comprehensive and molecularly informed comprehension of CO_2_ solvation in PIL systems,^52^ which serves as a primary motivator for the multi-structural comparison method used in this study.

**Fig. 2:**
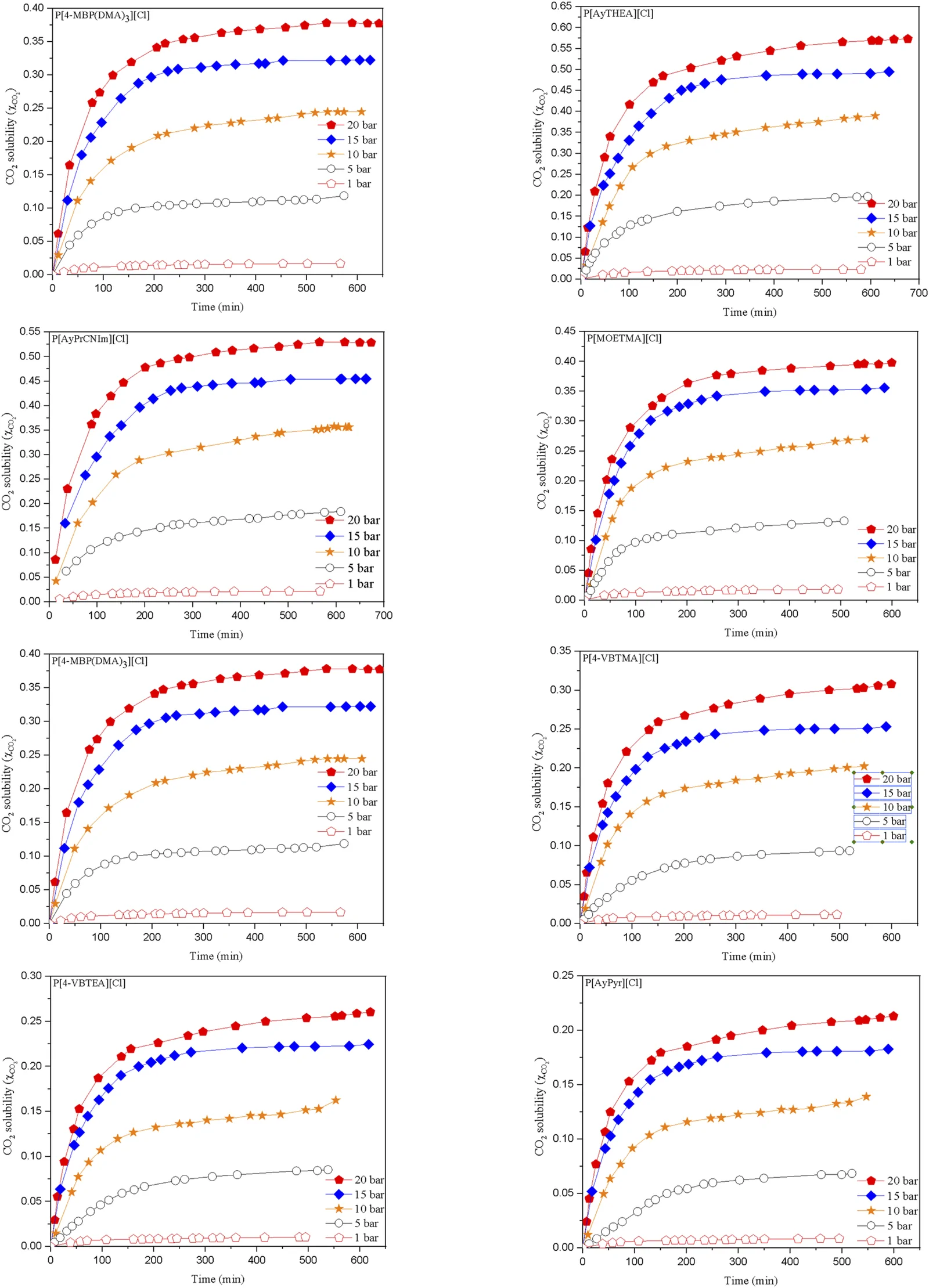
Effect of time on CO_2_ solubility in the synthesized PILs at 298.15 K and at pressures 1, 5, 10, 15, and 20 bar.

The time-dependent CO_2_ uptake in the PILs was described using a first-order saturation (pseudo-first-order) model:

1 *x*_CO_2__(*t*) = *x*_eq_(1 − *e*^−*kt*^)

where *x*_CO_2__(*t*) is the CO_2_ mole fraction at time *t* (minutes), *x*_eq_ is the equilibrium (saturation) mole fraction at a given pressure, and *k* (min^−1^) is the first-order rate constant governing the approach to equilibrium. The half-saturation time (*t*½ = ln(2)/*k*) represents the time required to reach 50% of the equilibrium capacity and provides an intuitive measure of absorption speed.^53^ These parameters allow for a thorough comparison of sorption kinetics and CO_2_ capacity among the synthesized PILs as functions of pressure and time.^54^ The three parameters along with the *R*^2^ values (coefficient of determination) were presented in the (SI) in the Tables S9–S12.

This functional form is often used to model diffusion-sorption processes, in which the rate of absorption is roughly proportional to the difference between instantaneous and equilibrium loadings. The model accurately described the experimental uptake curves, with *R* > 0.96 across all PILs and pressures. The apparent rate constant (*k*) is an effective parameter that integrates contributions from external mass transfer, internal diffusion, polymer relaxation, and instrumental response. It is not the same as the elementary reaction rate constant. The major goal of this kinetic study is to offer a consistent, quantitative foundation for evaluating the relative sorption rates of various PILs under comparable experimental settings, rather than assigning a particular chemical mechanism.^55,56^

The observed pressure-dependent gas capture in PILs is supported by the increase of *x*_eq_ with CO_2_ pressure across all studied PILs.^15,57^ P[4-VBPDMA-TMA][Cl] and P[AyTHEA][Cl] showed the best CO_2_ capture capacity at 20 bar. The improved CO_2_ capture capacity may be due to the improved CO_2_-cation interactions *via* van der Waals and Lewis acid–base interactions might be due to their quaternary ammonium and ethoxy side chains.^58^ The reduced equilibrium capacities of P[AyPyr][Cl] and P[4-VBTEA][Cl] might be due to less favorable electrostatic interactions and the limited free volume provided by their polymer backbones. The reduced *x*_eq_ values at 1 bar for all synthesized PILs demonstrate the significant pressure dependency of CO_2_ capture. Throughout the PILs literature, this conduct has been verified.^59,60^

The investigated PILs exhibited higher k values at 20 bar compared to intermediate pressures, which corresponds to results reported by Carlisle *et al.*^61^ and Bara *et al.*,^62^ who attributed the effect to a plasticization of the ionic polymer matrix caused by CO_2_ that reduces diffusion resistance. A number of PILs, including P[AyPyr][Cl], P[4-VBTEA][Cl], and P[4-VBTMA][Cl], showed noticeably delayed absorption at 5 bar, which is consistent with the findings of Tang *et al.*^63^ (Tang ascribed it to the competing interaction between chain mobility and CO_2_ capture). The time-based CO_2_ capture profiles of the studied PILs demonstrate a rapid initial increase, which is subsequently followed by a gradual transition to equilibrium. At 20 bar, the most remarkable rate of absorption was detected in P[4-VBPDMA-TMA][Cl] and P[AyTHEA][Cl]. These two PILs can be ideal for industrial applications because of their high capacity and rapid absorption.^64^ The *R*^2^ values (coefficient of determination) for all of these PILs were more than 0.96, indicating that the first-order saturation model well describes the kinetics of CO_2_ uptake.

#### 3.3.2 Impact of cations on the CO_2_ solubility

The most important factors that govern the CO_2_ capture efficacy of PILs are the structural factors such as; the nature of the cationic head group, the chemistry of attached functional groups, the structure of the polymer backbone, charge density, fractional free volume, and chain conformational flexibility.^65^ Given that all eight PILs studied in this comparison possess the same anion, the study focuses on the cationic structural framework as the principal variable influencing CO_2_ capture efficacy.^22,66^

PILs have significantly greater CO_2_ capture capacity and rapid sorption/desorption rates compared to their monomeric ILs equivalents.^59,66^ The cation of the PIL significantly influences CO_2_ sorption, as reported by the superior CO_2_ solubility in ammonium-type cations relative to imidazolium-type cations when the anion remains constant. The length of the alkyl chain and the steric shield of the cation impact the fractional free volume of the polymer, therefore influencing the CO_2_ sorption capabilities of the PILs as reported.^67^ In general, the sorption capacity follows the sequence of ammonium > pyridinium > phosphonium > imidazolium, as stated by Tang *et al.*,^60^ however this generalization requires modification due to unique functional group changes that may enhance or diminish sorption capacity of individual PIL relative to this standard order. Moreover, the sequence reported by Tang *et al.* reported the sequence is a good general trend for PILs with comparable backbones and anions, while the present results demonstrate that strategic functionalization of the cationic side chains can substantially modify this order. The findings of Tang are not contradicted by the deviations in this work; rather, they emphasize that cation identity is merely one of several factors that regulate CO_2_ sorption in PILs.^1^ The deviation of the results of the present PILs are due to several structural and physicochemical factors that override the “standard” cation order. The main factors that cause the deviation are the effect of the functional-group on charge density and polarity, the polymer backbone and morphology. Incorporating polar or basic functional groups (*e.g.*, hydroxyl, ether, amine, carbonyl) to pyridinium, phosphonium, or imidazolium cations might improve CO_2_-polymer interactions (dipole–quadrupole, hydrogen bonding), increase local free volume or microvoids for CO_2_.^1,2^ The rigidity level of the backbone, the density of cross-linking, and the pore architecture (such as mesoporous *vs.* dense) influence the free volume, diffusivity, and availability of ionic sites.^1,2^ As a results, the Tang sequence should be viewed as a baseline trend for structurally simple, closely related PILs (same backbone, similar anions, no special functional groups).

The capture of CO_2_ in the prepared PILs at temperature 298.15 K and pressures 1, 5, 10, 15 and 20 bar are measured and the time-dependence of CO_2_ uptake by the studied ILs is reported in Table S1–S8 in SI and shown in Fig. 3.

**Fig. 3:**
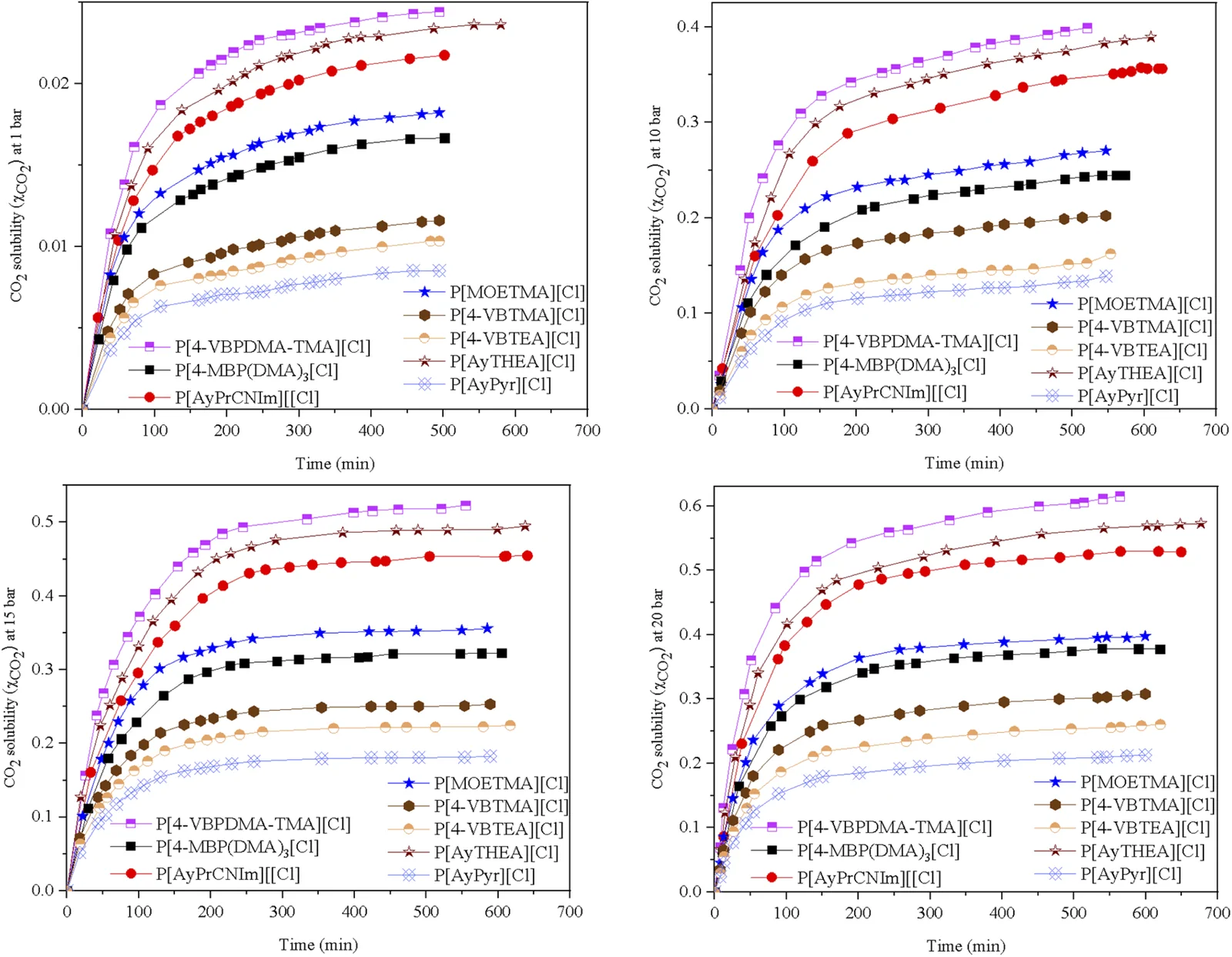
Effect of cation on CO_2_ capture efficiency in the prepared PILs at 1, 10, 15, and 20 bar.

Table 1 summarized the CO_2_ capture in the prepared PILs as a mole fraction of CO_2_ (*x*_CO_2__). The results demonstrated that the efficiency of CO_2_ capture at each of the investigated pressure in the synthesized PILs follow the order; P[4-VBPDMA-TMA][Cl] > P[AyTHEA][Cl] > P[AyPrCNIm][Cl] > P[MOETMA][Cl] > P[4-MBP(DMA)_3_][Cl] > P[4-VBTMA][Cl] > P[4-VBTEA][Cl] > P[AyPyr][Cl]. P[4-VBPDMA-TMA][Cl] has highest CO_2_ capture efficiency among the synthesized PILs, that could be attributable to its distinctive multifunctional CO_2_ interaction structure.^15^ The phosphino group in known to have the ability to impart a lone pair on phosphorus enabling contact with CO_2_*via* donor–acceptor interactions, while the two dimethylamino groups provide supplementary Lewis basic nitrogen sites, and the trimethylammonium group stabilizes the anion.^15,68^ The inflexible aromatic structure may diminish fractional free volume and diffusion rates, hence partly limiting kinetic performance.

**Table 1:** CO_2_ capture in the prepared PILs at 1, 5, 10, 15, and 20 bar as a mole fraction of CO_2_ (*x*_CO_2__) at 298.15 to 358.15 K

*T*/K	*P* (bar)	CO_2_ mol fraction							
		P[4-VBPDMAMA][Cl]	P[AyTHEA][Cl]	P[AyPrCNIm][Cl]	P[MOETMA][Cl]	P[4-MBP(DMA)_3_][Cl]	P[4-VBT MA][Cl]	P[4-VB TEA][Cl]	P[AyPyr] [Cl]
298.15	1	0.0244	0.0236	0.0219	0.0182	0.0168	0.0116	0.0103	0.0085
	5	0.2519	0.1973	0.1842	0.1329	0.1188	0.0938	0.0854	0.0686
	10	0.4267	0.3895	0.3563	0.2705	0.2448	0.2023	0.1624	0.1390
	15	0.5234	0.4949	0.4547	0.3559	0.3226	0.2532	0.2246	0.1827
	20	0.6156	0.573	0.5285	0.4096	0.3775	0.3078	0.2605	0.2131
	1	0.0125	0.019	0.0063	0.0086	0.0136	0.0172	0.0164	0.0077
	5	0.0909	0.1873	0.0555	0.074	0.0999	0.1443	0.1445	0.0674
	10	0.1965	0.2822	0.110	0.1596	0.2151	0.2999	0.278	0.1309
	15	0.2578	0.3699	0.1454	0.2021	0.2776	0.3808	0.3628	0.1741
	20	0.2915	0.4344	0.1639	0.2367	0.3068	0.4300	0.4100	0.2003
328.15	1	0.0091	0.0109	0.0046	0.0063	0.0099	0.0121	0.0120	0.0056
	5	0.0781	0.1333	0.0463	0.0591	0.0801	0.1209	0.1170	0.0539
	10	0.1572	0.2188	0.0858	0.1273	0.1695	0.2391	0.2188	0.1054
	15	0.2109	0.2855	0.1092	0.1632	0.209	0.3028	0.2815	0.1370
	20	0.2285	0.3301	0.1263	0.1845	0.2398	0.3518	0.3276	0.1561
343.15	1	0.0077	0.0095	0.0038	0.0051	0.0098	0.0097	0.0101	0.0046
	5	0.0588	0.0902	0.0378	0.0483	0.0647	0.0841	0.0825	0.0440
	10	0.1247	0.1676	0.0661	0.0994	0.1347	0.1833	0.1752	0.0822
	15	0.1624	0.2156	0.0832	0.1244	0.1672	0.2286	0.2222	0.1044
	20	0.1735	0.2401	0.0964	0.1408	0.1883	0.2594	0.2506	0.1199
358.15	1	0.0056	0.0049	0.0028	0.0038	0.0073	0.0069	0.0074	0.0034
	5	0.0504	0.0699	0.0312	0.0414	0.0554	0.0688	0.0739	0.0377
	10	0.1026	0.1285	0.0511	0.0768	0.1042	0.1404	0.1417	0.0636
	15	0.134	0.1649	0.0658	0.0984	0.1323	0.1748	0.1804	0.0826
	20	0.1409	0.184	0.0749	0.1093	0.1475	0.1987	0.2055	0.0935

The presence of the three hydroxyl groups in the polycation of P[AyTHEA][Cl] is expected to enhance CO_2_ capturing^68,69^ compared to non-hydroxylated ammonium PILs, which might be due to hydrogen-bonding interactions with CO_2_ and the expected increase of the local dielectric environment around the CO_2_.^70,71^ Nevertheless, an increased number of hydroxyl groups may concurrently increases inter-chain hydrogen bonding and water affinity, which can increase the bulk viscosity and hindering mass transfer kinetics.^72^

The superior CO_2_ capture efficiency of P[AyPrCNIm][Cl] compared to unfunctionalized imidazolium PILs may be attributed to the incorporation of the nitrile group.^73,74^ Generally, imidazolium-based PILs have the lowest CO_2_ capture efficiency among common cation families in polystyrene-backbone PILs.^67^ The limitation of CO_2_ capture efficiency in imidazolium-based PILs is overcame in P[AyPrCNIm][Cl] by the incorporation of the nitrile group (–CN), which is known to enhance CO_2_ affinity which can be explained by the dipole–quadrupole interactions with the CO_2_ molecule.^63,73–75^ Moreover, the CN group is expected to affect the physicochemical properties of P[AyPrCNIm][Cl] by interacting with adjacent ions, as well as increasing hydrogen-bonding interactions, both of which enhance the cationic environment's affinity for polar solutes, including CO_2_.^76^

The CO_2_ capture efficiency of P[MOETMA][Cl] may be ascribed to its flexible backbone and ester-facilitated CO_2_ interactions.^60^ In contrast to P[4-VBTMA][Cl], which has an inflexible polystyrene–benzylic structure, P[MOETMA][Cl] has a two-carbon ester-linked ethylene spacer (–OCH_2_CH_2_-N^+^Me_3_) bonding the cationic trimethylammonium moiety to a flexible poly(methacrylate) backbone. The ester linkage in the spacer adds a carbonyl group (C=O), it is reported that CO_2_ interacts with the oxygen atom of ester carbonyl linkages by Lewis acid–base interactions, whereby the electrophilic carbon of CO_2_ interacts with the electron-rich carbonyl oxygen of the ester group^77–79^ indicating that ester carbonyl groups serve as secondary CO_2_ interaction sites in PIL structures.

Phosphonium-based PILs known to have lower CO_2_ capture efficiency relative to ammonium analogues in terms of cation contribution. However, the tris(dimethylamino) substitution on prepared PIL (P[4-MBP(DMA)_3_][Cl]) introduces a substantial secondary effect (the dimethylamino nitrogen atoms and other Lewis basic nitrogen-containing moieties increase in the number of such interaction points and directly increases CO_2_ capture capacity in PILs) which might enhances the physical anion–CO_2_ interaction with an additional contribution. Additionally, molecular dynamics simulations of functionalized phosphonium-based PILs showed that when cations are functionalized with Lewis basic groups, the absorption energy and CO_2_ affinity are much higher than in unfunctionalized phosphonium analogues; the total uptake is determined by fractional free volume.^68,69^

P[4-VBTMA][Cl] is the direct structural analogue of P[4-VBTEA][Cl], differing only in the replacement of three methyl groups with three ethyl groups on the quaternary nitrogen. The relativley high CO_2_ capture efficiency of P[VBTMA][Cl] relative to P[VBTEA][Cl] is attributed to its more compact head group (trimethylammonium rather than triethylammonium) and hence high charge-density cationic site with low steric hindrance of the anion thus, maintaining ideal electrostatic accessibility for CO_2_ integration.^80^ The three ethyl chains increases the steric bulk around the nitrogen center, hence reducing the electrostatic accessibility of the anion to CO_2_ and lowering localized charge density.^63,81^ Nevertheless, longer ethyl chains are recognized to increase fractional free volume by disturbing compact chain packing, which may somewhat reduce the electronic disadvantage *via* improved CO_2_ capture efficiency.^81^

The P[AyPyr][Cl] structure exhibits an aromatic character of the pyridinium ring, which is known to have ability to delocalizes the positive charge throughout the ring system.^43^ This delocalization diminishes the localized electrostatic attraction for the anion, thereby weakening the anion–cation interaction and indirectly facilitating CO_2_ capture efficiency of the P[AyPyr][Cl] offering an additional geometrically based absorption route that is not present in aliphatic ammonium cations. In accordanec with Zhang *et al.* investigation, the CO_2_ capture efficiency of several PILs revealed that pyridinium PILs ranked second to ammonium in CO_2_ uptake performance.^82^ The adaptable polyallyl backbone of P[AyPyr][Cl] provides superior mobility compared to aromatic backbone counterparts, hence it may improve CO_2_ diffusivity and absorption kinetics.^83,84^

The results showed that the bifunctional and multi-centre cations yield the highest CO_2_ affinity. P[4-VBPDMA-TMA][Cl], bearing both phosphino and ammonium cationic centres with additional NMe_2_ Lewis basic sites, showed the highest CO_2_ capture efficiency in this series might be due to cooperative engagement of multiple interaction sites with CO_2_.^85^ Moreover, the nitrile group in P[AyPrCNIm][Cl] and the hydroxyethyl groups in P[AyTHEA][Cl].^15,71^ each elevate their PIL's CO_2_ capture efficiency substantially above what the cation identity would predict from the general ranking (ammonium > pyridinium > phosphonium > imidazolium).^86^ In addition, the PIL with polymethacrylate backbones (P[MOETMA][Cl]) exhibited faster absorption kinetics relative to rigid polystyrene-backbone analogues.^71^ The compact ammonium head groups (trimethyl amine cation in [VBTMA][Cl]) outperform sterically encumbered analogues (triethyl amine cation in [VBTEA][Cl]) in CO_2_ capture capacity, reflecting the dominant influence of localised charge density over free-volume effects in the physical absorption regime.^87^

#### 3.3.3 Henry's constant and dual-mode sorption (DMS) model

The Henry's Law constant has become the standard thermodynamic parameter for comparing CO_2_ capture performance across structurally varied IL systems, replacing the previous practice of reporting mass uptake or mole fraction solubility at a single pressure.^15,82,88^ The use of Henry's Law for interpreting CO_2_ capture in PILs requires several significant modifications and considerations compared to their monomeric ILs counterparts.^63,65^ The Henry's Law constant is usually expressed in the dual-mode sorption (DMS) model for solid PILs. This model divided the total CO_2_ sorption into two parts, which were a Langmuir-type saturation part (occupying pre-existing microvoids or glassy-state holes) and a Henry's Law dissolution part (physical absorption into the rubbery polymer matrix).^89,90^

Before applying the DMS model, the isotherms' nonlinearity must be verified using the ratio test that determines whether *x*_CO_2__/*P* is constant (Henry's Law) or decreasing (Langmuir behavior).^89,90^Table 2 reported the *x*_CO_2__/*P* ratio which verified that the isotherm is completely non-linear and that basic Henry's Law is inadequate across the entire pressure range. Consequently, the DMS model is essential.

**Table 2:** *x*_CO_2__/*P* ratio for the studied PILs at 298.15 K

Temperature (K)	Pressure (bar)	*x*_CO_2__/*P*							
		P[4-VBPDMA-TMA][Cl]	P[AyTHEA][Cl]	P[AyPrCNIm][Cl]	P[MOETMA][Cl]	P[4-MBP(DMA)_3_][Cl]	P[4-VBTMA][Cl]	P[4-VBTEA][Cl]	P[AyPyr][Cl]
298.15	1	0.0244	0.0236	0.0219	0.0182	0.0168	0.0116	0.0103	0.0085
	5	0.0504	0.0395	0.0368	0.0266	0.0238	0.0188	0.0171	0.0137
	10	0.0427	0.0390	0.0356	0.0271	0.0245	0.0202	0.0162	0.0139
	15	0.0349	0.0330	0.0303	0.0237	0.0215	0.0169	0.0150	0.0122
	20	0.0308	0.0287	0.0264	0.0205	0.0189	0.0154	0.0130	0.0107
313.15	1	0.0125	0.0190	0.0063	0.0086	0.0136	0.0172	0.0164	0.0077
	5	0.0182	0.0375	0.0111	0.0148	0.0200	0.0289	0.0289	0.0135
	10	0.0197	0.0282	0.0110	0.0160	0.0215	0.0300	0.0278	0.0131
	15	0.0172	0.0247	0.0097	0.0135	0.0185	0.0254	0.0242	0.0116
	20	0.0146	0.0217	0.0082	0.0118	0.0153	0.0215	0.0205	0.0100
328.15	1	0.0091	0.0109	0.0046	0.0063	0.0099	0.0121	0.0120	0.0056
	5	0.0156	0.0267	0.0093	0.0118	0.0160	0.0242	0.0234	0.0108
	10	0.0157	0.0219	0.0086	0.0127	0.0170	0.0239	0.0219	0.0105
	15	0.0141	0.0190	0.0073	0.0109	0.0139	0.0202	0.0188	0.0091
	20	0.0114	0.0165	0.0063	0.0092	0.0120	0.0176	0.0164	0.0078
343.15	1	0.0077	0.0095	0.0038	0.0051	0.0098	0.0097	0.0101	0.0046
	5	0.0118	0.0180	0.0076	0.0097	0.0129	0.0168	0.0165	0.0088
	10	0.0125	0.0168	0.0066	0.0099	0.0135	0.0183	0.0175	0.0082
	15	0.0108	0.0144	0.0055	0.0083	0.0111	0.0152	0.0148	0.0070
	20	0.0087	0.0120	0.0048	0.0070	0.0094	0.0130	0.0125	0.0060
358.158	1	0.0056	0.0049	0.0028	0.0038	0.0073	0.0069	0.0074	0.0034
	5	0.0101	0.0140	0.0062	0.0083	0.0111	0.0138	0.0148	0.0075
	10	0.0103	0.0129	0.0051	0.0077	0.0104	0.0140	0.0142	0.0064
	15	0.0089	0.0110	0.0044	0.0066	0.0088	0.0117	0.0120	0.0055
	20	0.0070	0.0092	0.0037	0.0055	0.0074	0.0099	0.0103	0.0047

The effective Henry's Law constant across the dual-mode framework is obtained from the initial slope of the *x*_CO_2__*versus* pressure isotherm as the pressure approaches zero using the following equation:^89,90^2
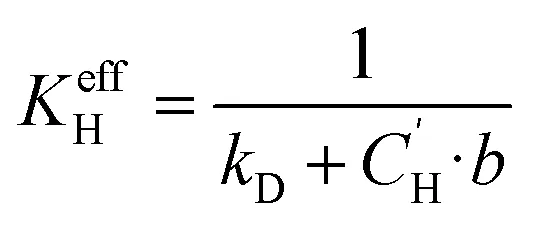
where *K*^eff^_H_ is effective Henry's Law constant, *k*_D_ is Henry's dissolution coefficient 
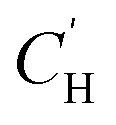
, is Langmuir hole-saturation capacity, and *b* is Langmuir affinity constant.

*k*_D_ (mol frac per bar) represents the dissolution into the rubbery/mobile polymer matrix *via* physical absorption; scales linearly with pressure 
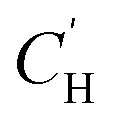
, (mol CO_2_/mol PIL) represents the maximum CO_2_ that can occupy pre-existing microvoids (Langmuir sites) in the glassy PIL matrix; pressure-independent at saturation), and *b* (bar^−1^) is defined as the strength of CO_2_ interaction with Langmuir sites; higher *b* = stronger site affinity).

*k*_D_ Values was constrained to zero in the reported dual-mode-sorption fits. This constraint was adopted because the chloride-based PILs behaved as glassy, ionically crosslinked-like materials under the measurement conditions, and their strongly nonlinear isotherms (Table 2) were dominated by saturation of pre-existing microvoids. Accordingly, the Langmuir contribution provides the principal description of CO_2_ uptake. For all the studied PILs in this series, as the isotherm has a pronounced Langmuir characteristic with an insignificant linear Henry dissolution component at the observed pressures; hence, the initial estimated values have been fixed as; 
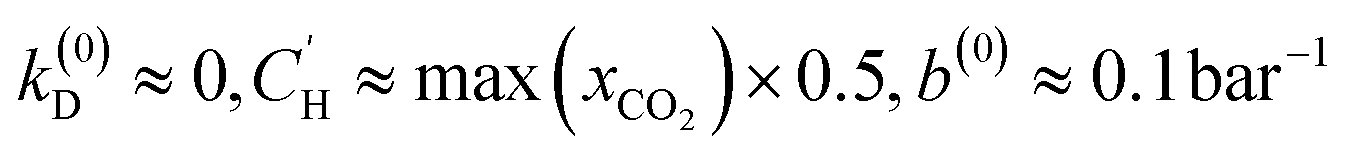
.^91,92^ The *K*^eff^_H_along with the DMS fitted parameters were calculated and reported in Table 3.

**Table 3:** Dual-mode sorption model fitted parameters and effective Henry's Law constants for studied PILs at 298.15 K^a^

PIL	*T*/K	*k*_D_ (mol frac per bar)	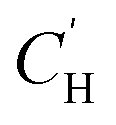 (mol CO_2_/mol PIL)	*b* (bar^−1^)	*K* ^eff^_H_ (bar)	*K* ^(1bar)^_H_ (bar)	*R* ^2^	RMSE
P[4-VBPDMA-TMA][Cl]	298.15	0.000	1.2204	0.0512	16.02	40.98	0.9933	0.0171
P[AyTHEA][Cl]		0.000	1.3865	0.0363	19.87	42.37	0.9929	0.0169
P[AyPrCNIm][Cl]		0.000	1.2763	0.0363	21.59	45.66	0.9940	0.0143
P[MOETMA][Cl]		0.000	1.1010	0.0307	29.61	54.95	0.9932	0.0119
P[4-MBP(DMA)_3_][Cl]		0.000	1.0836	0.0275	33.57	59.52	0.9942	0.0101
P[4-VBTMA][Cl]		0.000	0.9010	0.0263	42.18	86.21	0.9917	0.0098
P[4-VBTEA][Cl]		0.000	0.7728	0.0262	49.48	97.09	0.9957	0.0060
P[AyPyr][Cl]		0.000	0.5929	0.0289	58.42	117.65	0.9939	0.0058
P[4-VBPDMA-TMA][Cl]	313.15	0.000	0.7927	0.0305	20.14	80.00	0.9889	0.0110
P[AyTHEA][Cl]		0.000	0.8260	0.0518	25.49	52.63	0.9931	0.0122
P[AyPrCNIm][Cl]		0.000	0.4148	0.0341	28.82	158.73	0.9916	0.0053
P[MOETMA][Cl]		0.000	0.6437	0.0300	34.21	116.28	0.9900	0.0084
P[4-MBP(DMA)_3_][Cl]		0.000	0.7565	0.0362	36.80	73.53	0.9858	0.0132
P[4-VBTMA][Cl]		0.000	1.0375	0.0371	45.33	58.14	0.9887	0.0163
P[4-VBTEA][Cl]		0.000	0.9799	0.0375	52.39	60.98	0.9925	0.0125
P[AyPyr][Cl]		0.000	0.5300	0.0314	62.36	129.87	0.9941	0.0054
P[4-VBPDMA-TMA][Cl]	328.15	0.000	0.5584	0.0371	27.10	109.89	0.9866	0.0095
P[AyTHEA][Cl]		0.000	0.7047	0.0447	32.10	91.74	0.9937	0.0090
P[AyPrCNIm][Cl]		0.000	0.2885	0.0400	34.75	217.39	0.9937	0.0035
P[MOETMA][Cl]		0.000	0.4754	0.0333	42.40	158.73	0.9870	0.0076
P[4-MBP(DMA)_3_][Cl]		0.000	0.5674	0.0381	40.58	101.01	0.9878	0.0094
P[4-VBTMA][Cl]		0.000	0.8588	0.0357	49.10	82.64	0.9920	0.0110
P[4-VBTEA][Cl]		0.000	0.7853	0.0367	56.33	83.33	0.9944	0.0086
P[AyPyr][Cl]		0.000	0.3840	0.0356	66.48	178.57	0.9921	0.0049
P[4-VBPDMA-TMA][Cl]	343.15	0.000	0.4045	0.0407	29.20	129.87	0.9818	0.0085
P[AyTHEA][Cl]		0.000	0.5236	0.0445	37.86	105.26	0.9914	0.0079
P[AyPrCNIm][Cl]		0.000	0.2028	0.0462	40.05	263.16	0.9943	0.0025
P[MOETMA][Cl]		0.000	0.3286	0.0393	48.11	196.08	0.9880	0.0055
P[4-MBP(DMA)_3_][Cl]		0.000	0.4256	0.0417	44.77	102.04	0.9883	0.0072
P[4-VBTMA][Cl]		0.000	0.6354	0.0362	51.13	103.09	0.9856	0.0111
P[4-VBTEA][Cl]		0.000	0.6135	0.0363	59.48	99.01	0.9876	0.0100
P[AyPyr][Cl]		0.000	0.2705	0.0410	69.92	217.39	0.9933	0.0034
P[4-VBPDMA-TMA][Cl]	358.15	0.000	0.3130	0.0449	34.08	178.57	0.9806	0.0072
P[AyTHEA][Cl]		0.000	0.4016	0.0444	44.05	204.08	0.9895	0.0067
P[AyPrCNIm][Cl]		0.000	0.1506	0.0507	45.08	357.14	0.9934	0.0021
P[MOETMA][Cl]		0.000	0.2362	0.0453	55.75	263.16	0.9903	0.0038
P[4-MBP(DMA)_3_][Cl]		0.000	0.3144	0.0464	49.68	136.99	0.9921	0.0046
P[4-VBTMA][Cl]		0.000	0.4594	0.0398	54.42	144.93	0.9882	0.0077
P[4-VBTEA][Cl]		0.000	0.4719	0.0400	62.21	135.14	0.9918	0.0066
P[AyPyr][Cl]		0.000	0.1953	0.0473	72.65	294.12	0.9934	0.0026

a The results show that the *K*^eff^_H_ values follow the order P[4-VBPDMA-TMA][Cl] < P[AyTHEA][Cl] < P[AyPrCNIm][Cl] < P[MOETMA][Cl] < P[4-MBP(DMA)_3_][Cl] < P[4-VBTMA][Cl] < P[4-VBTEA][Cl] < P[AyPyr][Cl].

For the investigated PILs, the integration of *k*_D_ to zero is a physically significant and reliable finding that illustrates the mostly glassy nature of these chloride-based PIL materials under the experimental conditions as reported.^91,92^ Due to the strong coulombic ion-pair interactions between the cationic backbone and the [Cl^−^] anion, [Cl^−^]-based PILs are known to have relatively high glass transition temperatures.^65^ As a result, these PILs behave primarily as glassy materials in which chain mobility is reduced and CO_2_ sorption proceeds almost through occupation of pre-existing microvoid Langmuir sites (
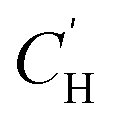
, *b* contribution) rather than Henry-type dissolution into a rubbery matrix.^91^ This result is in line with that reported for PILs based on fluoride and chloride.^75,93^

The fitted 
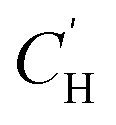
 values of the examined PILs vary from 0.5929 to 1.3865 mol fraction, indicating the total capacity of microvoid Langmuir sites accessible for CO_2_ accommodation inside each PIL matrix.^63^
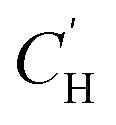
 is known to be influenced by the fractional free volume of the glassy polymer, where more disorder, bulkiness, or asymmetry in repeat unit structures results in larger interchain void space and thus elevated 
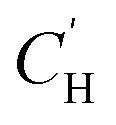
 The highest 
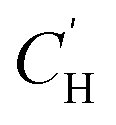
 values found were 1.3865 and 1.2204 mol fraction reported for P[AyTHEA][Cl], and P[4-VBPDMA-TMA][Cl] respectively, both featuring large, sterically demanding cationic head groups which are known to impede efficient chain packing and create significant interchain free volume.^32,93^ In contrast, the lowest 
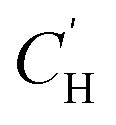
 is noted for P[AyPyr][Cl] (0.5929 mol fraction), as its more planar pyridinium cation have the ability to achieves more effective packing with less internal empty space.^63^

The thermodynamic intensity of CO_2_ interaction with the Langmuir microvoids sites in each PIL is measured by the affinity constant *b*.^93,94^ The bifunctional phosphino-ammonium PIL (P[4-VBPDMA-TMA][Cl]) has a larger *b* indicating its dual-center cation's stronger electrostatic and Lewis acid–base interaction capacity, which allows engaging CO_2_*via* multiple donor–acceptor interactions.^95^ The Lewis basicity of the cationic environment is known to govern the relationship between *b* and molecular structure: PILs with more electron-rich cationic centers donate more electron density to the [Cl^−^] anion, increasing its effective Lewis basicity toward CO_2_ and consequently raising the affinity constant *b*.^65,95^

For the studied PILs, the *K*^eff^_H_ is much lower than the 1-bar estimates (*K*^(1bar)^_H_). The ratio *K*^(1bar)^_H_)/*K*^eff^_H_ ranges from 1.99 to 2.56. This estimation invalidates the basic Henry's Law as a quantitative indicator of CO_2_ affinity over the whole pressure range by confirming that substantial Langmuir-site filling occurs even at 1 bar.^32,75^ The combined effect of the *b* and 
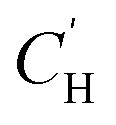
 on CO_2_ sorption is directly reflected in the *K*^eff^_H_ values. Because of its exceptionally high *b* value (indicates strong multifunctional cation–CO_2_ interactions) and its large 
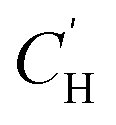
 value (indicates significant interchain free volume produced by the bulky bifunctional cation) the bifunctional P[4-VBPDMA-TMA][Cl] achieved the highest CO_2_ affinity.^95^ This result corresponds with the comprehensive principle indicating that affinity increases with the structural complexity of cations.^59^

#### 3.3.4 Enthalpy, entropy and gibbs free energy

The solubility parameters have been demonstrated by the thermodynamic excess functions: standard enthalpy (Δ*H*^0^_sol_), entropy (Δ*S*^0^_sol_), and Gibbs free energy (Δ*G*^0^_sol_), in general, negative values in excess Δ*G*^0^_sol_ often indicate the production of chemical complexes reflects the spontaneity of gas dissolution and the stability of the system's phases.^96^ The Δ*S*^0^_sol_ and Δ*H*^0^_sol_ indicates the degree of order and the strength of interaction between the liquid and the gas respectively.^40,97,98^ The efficiency of CO_2_ capture in PIL systems is determined by the sign and magnitude of thermodynamic variables such as Henry's Law constant (DMS model), Gibbs free energy, enthalpy, and entropy. Smaller positive Δ*G*^0^ values indicate higher CO_2_ affinity.^99^ Furthermore, eqn (3) is used to calculate Δ*G*^0^_sol_ using the standard pressure *P*^0^ = 1.01325 bar and Henry's Law constant (DMS model).^6^3
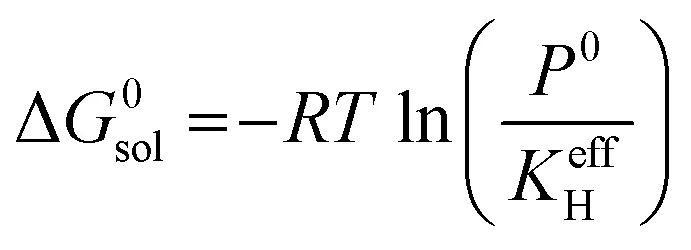


Δ*H*^0^_sol_ can be used as a constant and related to the effective Henry's law constant (DMS) at infinite dilution using the equation:4
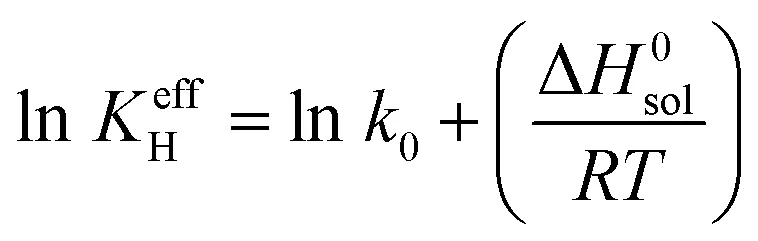


However, there are other cases, in which Δ*H*^0^_sol_ is temperature dependent and is a variant, thus can be estimated from equation:.^100^5
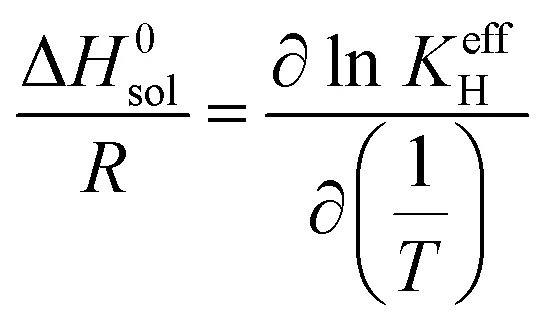


The change in molar enthalpy upon gas solubility is obtained by plotting the natural logarithm of the calculated effective Henry constant (DMS) ln *K*^eff^_H_*versus*
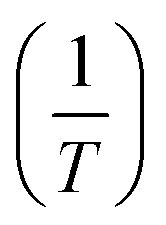
 and the curve was fitted with the following equation:^100^6
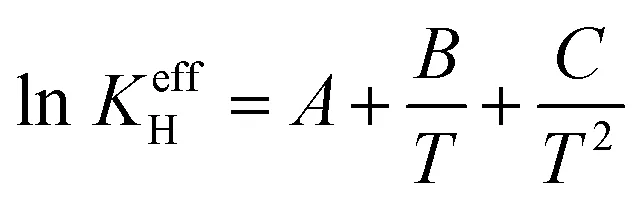
where, *A*, *B* and *C* are correlation coefficients. The eqn (4) and (5), were used to estimate the Δ*H*^0^_sol_ using the following equation:^100^7
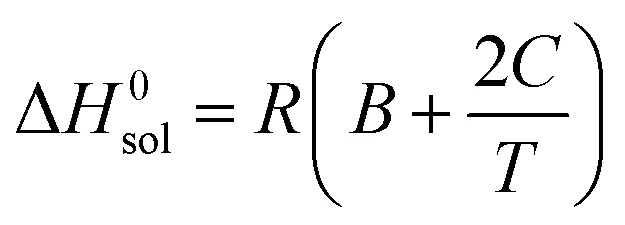


The Δ*S*^0^_sol_ was calculated using the following equation:^100^8
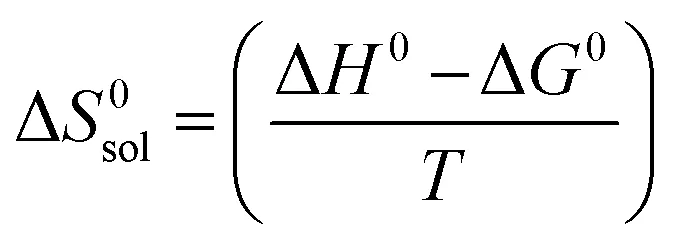


The thermodynamic properties (Δ*G*^0^_sol_, Δ*H*^0^_sol_, Δ*S*^0^_sol_) for CO_2_ capture in the synthesized PILs are calculated and presented in Table 4. It is noted that the studied PILs has positive values of Δ*G*^0^_sol_ throughout the temperature range examined (298.15–358.15 K). This favorable Δ*G*^0^_sol_ requires thorough and accurate thermodynamic analysis.^41,101^ This doesn't indicate that CO_2_ dissolution is thermodynamically unfavorable; rather, it confirms that the standard state chemical potential of CO_2_ in the PIL phase is greater than that in the reference gas-phase standard state, which is consistent with the physical reality that CO_2_ is more stable in the gas phase than in the PIL at standard pressure.^41,101,102^ Nevertheless, the Gibbs energy increases systematically with temperature, demonstrating that elevated temperature reduces the thermodynamic affinity of the PILs for CO_2_. This behavior agrees with the generally observed reduction in CO_2_ solubility in ionic liquids and poly(ionic liquid)s at increasing temperature.

**Table 4:** Thermodynamic properties (Δ*G*^0^_sol_, Δ*H*^0^_sol_, Δ*S*^0^_sol_) for CO_2_ capture in the synthesized PILs

Thermodynamic property	PILs	*T*/K				
		298.15	313.15	328.15	343.15	358.15
Δ*G*^0^_sol_ (kJ mol^−1^)	P[4-VBPDMA-TMA][Cl]	7.80	8.43	9.10	9.80	10.54
	P[AyTHEA][Cl]	5.37	6.34	7.47	8.36	9.28
	P[AyPrCNIm][Cl]	9.09	9.71	10.35	10.97	11.56
	P[MOETMA][Cl]	8.38	8.99	9.64	10.19	10.82
	P[4-MBP(DMA)_3_][Cl]	7.00	7.71	8.45	9.18	9.98
	P[4-VBTMA][Cl]	5.96	6.71	7.87	8.70	9.53
	P[4-VBTEA][Cl]	6.23	7.04	8.15	8.93	9.67
	P[AyPyr][Cl]	8.93	9.53	10.14	10.80	11.41
Δ*H*^0^_sol_ (kJ mol^−1^)	P[4-VBPDMA-TMA][Cl]	−4.31	−5.15	−5.92	−6.63	−7.27
	P[AyTHEA][Cl]	−16.11	−15.04	−14.07	−13.18	−12.37
	P[AyPrCNIm][Cl]	−3.79	−3.50	−3.22	−2.98	−2.75
	P[MOETMA][Cl]	−4.07	−3.88	−3.71	−3.55	−3.41
	P[4-MBP(DMA)_3_][Cl]	−6.90	−7.41	−7.87	−8.29	−8.68
	P[4-VBTMA][Cl]	−12.87	−12.51	−12.19	−11.89	−11.61
	P[4-VBTEA][Cl]	−13.90	−12.41	−11.05	−9.82	−8.68
	P[AyPyr][Cl]	−2.96	−3.25	−3.51	−3.74	−3.96
Δ*S*^0^_sol_ (J mol^−1^ K^−1^)	P[4-VBPDMA-TMA][Cl]	−40.62	−43.40	−45.80	−47.88	−49.73
	P[AyTHEA][Cl]	−72.03	−68.26	−65.63	−62.77	−60.46
	P[AyPrCNIm][Cl]	−43.20	−42.18	−41.37	−40.64	−39.96
	P[MOETMA][Cl]	−41.75	−41.09	−40.66	−40.05	−39.73
	P[4-MBP(DMA)_3_][Cl]	−46.59	−48.28	−49.73	−50.93	−52.10
	P[4-VBTMA][Cl]	−63.15	−61.39	−61.11	−59.99	−59.03
	P[4-VBTEA][Cl]	−67.50	−62.11	−58.53	−54.63	−51.25
	P[AyPyr][Cl]	−39.89	−40.80	−41.59	−42.40	−42.92

Moreover, the positive Δ*G*^0^_sol_ values (+5.37 to +11.56 kJ mol^−1^) correspond to an expected thermodynamic model for physical CO_2_ absorption in chloride-anion systems, where the weak anion–CO_2_ interaction results in low Henry's Law constant.^102,103^ The overall ranking of Δ*G*^0^_sol_at 298.15 K is: P[AyTHEA][Cl] < P[4-VBTMA][Cl] < P[4-VBTEA][Cl] < P[4-MBP(DMA)_3_][Cl] <P[4-VBPDMA-TMA][Cl] < P[MOETMA][Cl] < P[AyPyr][Cl] < P[AyPrCNIm][Cl] confirming internal thermodynamic consistency of the CO_2_ solubility data.^41,101,103,104^ As temperature rises, all studied PILs exhibit a consistent increase in Δ*G*^0^_sol_, indicating the exothermic dissolving process.^105,106^

All PILs exhibit positive Δ*H*^0^_sol_ values, indicating that CO_2_ dissolution is endothermic according to the applied convention. The largest enthalpy changes were obtained for P[4-VBPDMA-TMA][Cl] and P(4-VBTEA)[Cl], suggesting a comparatively large energetic requirement for the reorganization of the polymeric ionic-liquid matrix during CO_2_ incorporation. In contrast, P[AyPyCNm][Cl], P[MOETMA][Cl], P[4-VBPDMA-TMA][Cl], and P[AyPyr][Cl] exhibit smaller positive enthalpies, indicating a lower energetic value.

Δ*H*^0^_sol_ values of the studied PILs (−16.11 to −2.75 kJ mol^−1^) are consistent with the range reported for [BF_4_]-based ILs (approximately −9.5 to −12 kJ mol^−1^),^107^ for non-amine-functionalized PILs (−3 to −20 kJ mol^−1^) and for fluorinated imidazolium ILs (−11.6 to −14.7 kJ mol^−1^ at 298 K).^104,107,108^ Furthermore, the prepared PILs showed broader Δ*H*^0^_sol_ range than the representative values often reported for simple fluorinated ionic liquids. The most exothermic sample, P[AyTHEA][Cl], reaches approximately −16.11 kJ mol^−1^, while P[AyPyr][Cl] is only about −2.96 kJ mol^−1^. This broader range may result from polymer-specific effects such as restricted chain mobility, distribution of ionic sites along the backbone, or different CO_2_ sorption locations. These results are significantly well below the −80 kJ mol^−1^ level indicating the level of chemical absorption by carbamate production in amine-functionalized ILs,^84^ hence denying any considerable chemisorption contribution in these PILs systems.^53^ The calculated enthalpy values for the prepared PILs indicated predominantly physical sorption which simplifies desorption and cycling processes. PILs can be released by modifying pressure or temperature.^109,110^

The most exothermic enthalpy is recorded for P[AyPyr][Cl] and P[AyPrCNIm][Cl], which exhibit greater exothermic than the other six PILs, reflecting the multifaceted interactions present in these two PILs. Conversely, the PILs with simple unfunctionalized aromatic or aliphatic cations—P[AyTHEA][Cl], P[4-VBTMA][Cl], and P[4-VBTEA][Cl] demonstrate the least exothermic Δ*H*^0^_sol_values, indicating that CO_2_ absorption is predominantly influenced by weak van der Waals forces without specific functional group enhancement.^53,104^ The studied PILs showed strong, intermediate, and weak temperature dependence respectively. These results indicates a large negative, moderate negative, and zero heat capacity of dissolution respectively^106,112,104,95,111,53^ Moreover, the low-to-moderate Δ*H*^0^_sol_ values for the studied PILs are highly advantageous for energy-efficient regeneration: as the heat of desorption equals ∣Δ*H*^0^_sol_∣, these PILs will require lower regeneration energy compared to aqueous amine systems (∼80 kJ mol^−1^).^16,17^

Although the sorbent is polymeric, Δ*S*^0^_sol_ values (−72.03 to −39.73 J mol^−^ K^−1^) are similar to those reported for ILs based on bis(trifluoromethylsulfonyl)imide (−50 to 70 J mol^−1^ K^−1^),^17^ indicating that the CO_2_ ordering in the PIL matrix is the same magnitude as in conventional ILs.^111^ The highest entropy values are recorded for P[AyTHEA][Cl], P[4-VBEMA][Cl], and P[4-VBTMA][Cl], demonstrating that CO_2_ dissolution in these two PILs results in the highest degree of ordering, which aligns with their large, structurally complicated cations containing multiple specific interaction sites that provide significant orientational constraints on the dissolved CO_2_ molecule.^15,73,105,112^ The lowest negative entropy values are observed for (P[4-VBPDMA-TMA][Cl], P[MOETMA][Cl], and P[AyPrCNIm][Cl], indicating reduced ordering of CO_2_ in the PIL phase which is reported as a result of weaker and less structurally specific CO_2_-PIL interactions.^104,107,113^

## 4. Conclusion

The present study examined the CO_2_ capture efficacy of eight structurally varied chloride-based PILs derived from ammonium, phosphonium, imidazolium, and pyridinium cationic families, each functionalized with different chemical groups. Extensive gravimetric solubility studies using a high-precision magnetic suspension balance (MSB) over five temperatures (298.15–358.15 K) and five pressures (1–20 bar), in addition to kinetic modeling, DMS analysis, and thermodynamic characterization, yielded a comprehensive understanding of how the molecular structure of PIL influences CO_2_ capture behavior. The study found that multifunctional interaction sites provide a higher CO_2_ affinity than individual functional centers. The bifunctional PIL P[4-VBPDMA-TMA][Cl] had the highest CO_2_ capture capacity in the series while P[AyPyr][Cl] had the least amount of uptake. The effective Henry dissolution coefficient *K*^eff^_H_ approached zero for all studied PILs. Kinetic study using the first-order saturation model revealed that rate constants increased with pressure. P[4-VBPDMA-TMA][Cl] and P[AyTHEA][Cl] have high equilibrium capacity and fast capture kinetics at increased pressures, making them the most attractive PILs for pressure-swing CO_2_ capture. Thermodynamic analysis throughout the 298.15–358.15 K range revealed that CO_2_ dissolution in all investigated PILs is exothermic and accompanied by a negative entropy change. 
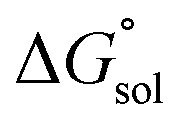
 values are positive and increase with temperature, demonstrating that CO_2_ dissolution becomes less favorable at elevated temperature under the selected standard-state convention. P[AyTHEA][Cl] exhibits the lowest 
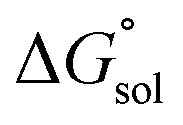
 values and the most negative Δ*H*^0^_sol_, suggesting the strongest apparent CO_2_ affinity among the investigated PILs. The negative Δ*S*^0^_sol_ values indicate increased ordering or restriction of CO_2_ in the PILs phase which is consistent with observations in the literature that CO_2_ solubility in PILs diminishes with temperature and that dissolution is typically entropically unfavorable and exothermic. The study proposes three key structure–activity concepts for designing next-generation CO_2_-capturing by PILs. Multifunctional cations with cooperative Lewis-basic sites enhance CO_2_ affinity *via* additive interactions, making bifunctional structural design the most effective technique for increasing absorption capacity. Incorporating polar functional groups, such as hydroxyethyl, nitrile, or ester carbonyl, significantly improves CO_2_ capture performance, exceeding the general ammonium > pyridinium > phosphonium > imidazolium cation-family ranking. This shows that targeted functionalization can overcome inherent cation-family limitations. Flexible polymer backbones and compact ammonium head groups promote rapid absorption kinetics and higher charge-density-driven CO_2_ interactions, respectively. This provides clear criteria for backbone and head-group selection in PIL synthesis.

## Conflicts of interest

There are no conflicts to declare.

## Supplementary Material

RA-016-D6RA05244D-s001

## Data Availability

The data that support our findings are available in this article and the supplementary information (SI). Supplementary information is available. See DOI: https://doi.org/10.1039/d6ra05244d.
