# Supplementary material for: Polar groups, charge density, and backbone flexibility as molecular determinants of CO2 capture efficiency and absorption kinetics of polymerized ionic liquids
Source: RSC Adv. 2026 Sep 28;16(45):58036–55. doi: 10.1039/d6ra05244d (PMC13618330; doi:10.1039/d6ra05244d)
Supplement: Supplementary file 1 [file RA-016-D6RA05244D-s001.pdf]

Supplementary Information (SI) for RSC Advances.
This journal is © The Royal Society of Chemistry 2026

Supplementary Information

Polar Groups, Charge Density, and Backbone Flexibility as Molecular Determinants of CO_2 Capture Efficiency and Absorption Kinetics of Polymerized Ionic Liquids

Ahmed Mohamed Abdelmagid[1], Abobakr Khidir Ziyada [1,2], Abdalla Ahmed Elbashir[3*], Zakaria A. Salih[4]*

[1]Department Chemical Engineering and Chemical Technology, Faculty of Engineering and Technology, University of Gezira, Wad Medani, 21113, Sudan

[2] General Studies Department, Jubail Industrial College, Jubail Industrial City 31961, Saudi Arabia

[3]Department of Chemistry, College of Science, King Faisal University, P.O.Box 400, Al-Ahsa, 31982, Saudi Arabia.

[4]Research and Training Station, King Faisal University, P.O.Box 400, Al-Ahsa, 31982, Saudi Arabia.

*All correspondence should be addressed to Ahmed Mohamed Abdelmagid and Abdalla Ahmed Elbashir

* Authors whom all correspondence should be addressed.

Email: bahooti@gmail.com

E mail: aaeahmed@kfu.edu.sa

Supplementary Information

1. NMR results:

The nuclear magnetic resonance (NMR), elemental analysis (actual vs. theoretical percent), mass fraction of water, mass fraction of halide (chloride), and purity data for all the prepared ILs are shown as follows:

P[AyTHEA][Cl]; δ_H(600 MHz; DMSO): = 8.10 (N-CH_2-*CH*-CH), 4.37 (N-CH_2-CH_2O*H*), 3.89 (N-CH_2-CH), 3.68 (N-CH_2-CH_2OH), 2.99 (N-CH_2-CH_2OH). Elemental analysis: % actual C, 49.47; H, 10.19; N, 5.48 $(C_9H_{20}NClO_3)_n$. % theoretical C, 49.68; H, 10.01; N, 5.79. Mass fraction of water 246×10^{-6}.

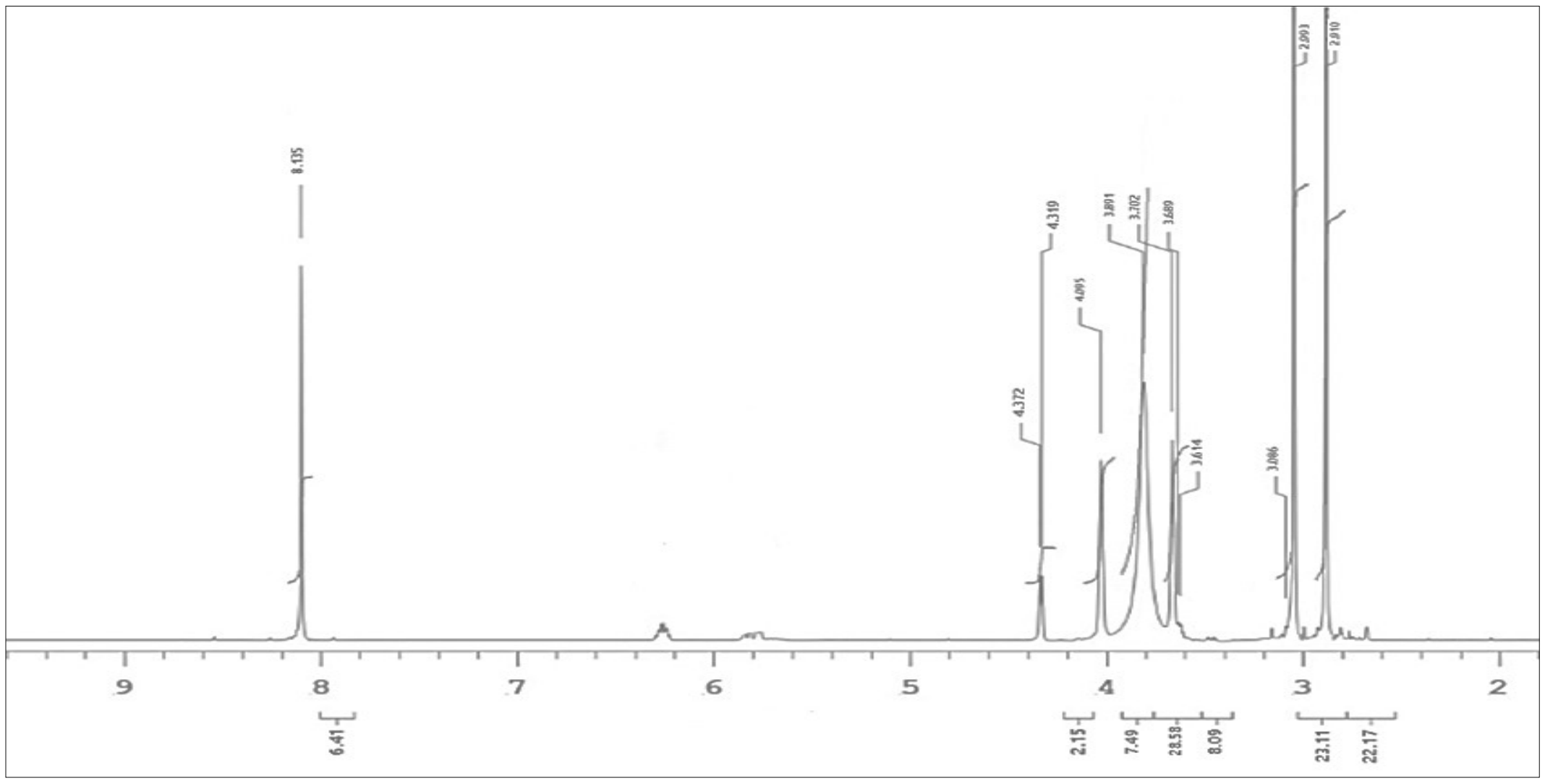

Figure S. 1 ^{1}HNMR spectra for P[AyTHEA][Cl]

P[4-VBTMA][Cl]; δ_H(600 MHz; DMSO): = 7.37 (CH_2-C(*CH)$_2$* Ar), 6.56 (CH-C (*CH)$_2$* Ar), 4.93 (N-CH_2-C), 3.20 (N-$(CH_3)_3$), 3.09 (C-*CH*-CH_2), 1.60 (C-CH-CH_2). Elemental analysis: % actual C, 67.01; H, 9.59; N, 6.42 $(C_{12}H_{20}NCl)_n$. % theoretical C, C, 67.43; H, 9.43; N, 6.55. Mass fraction of water 215×10^{-6}.

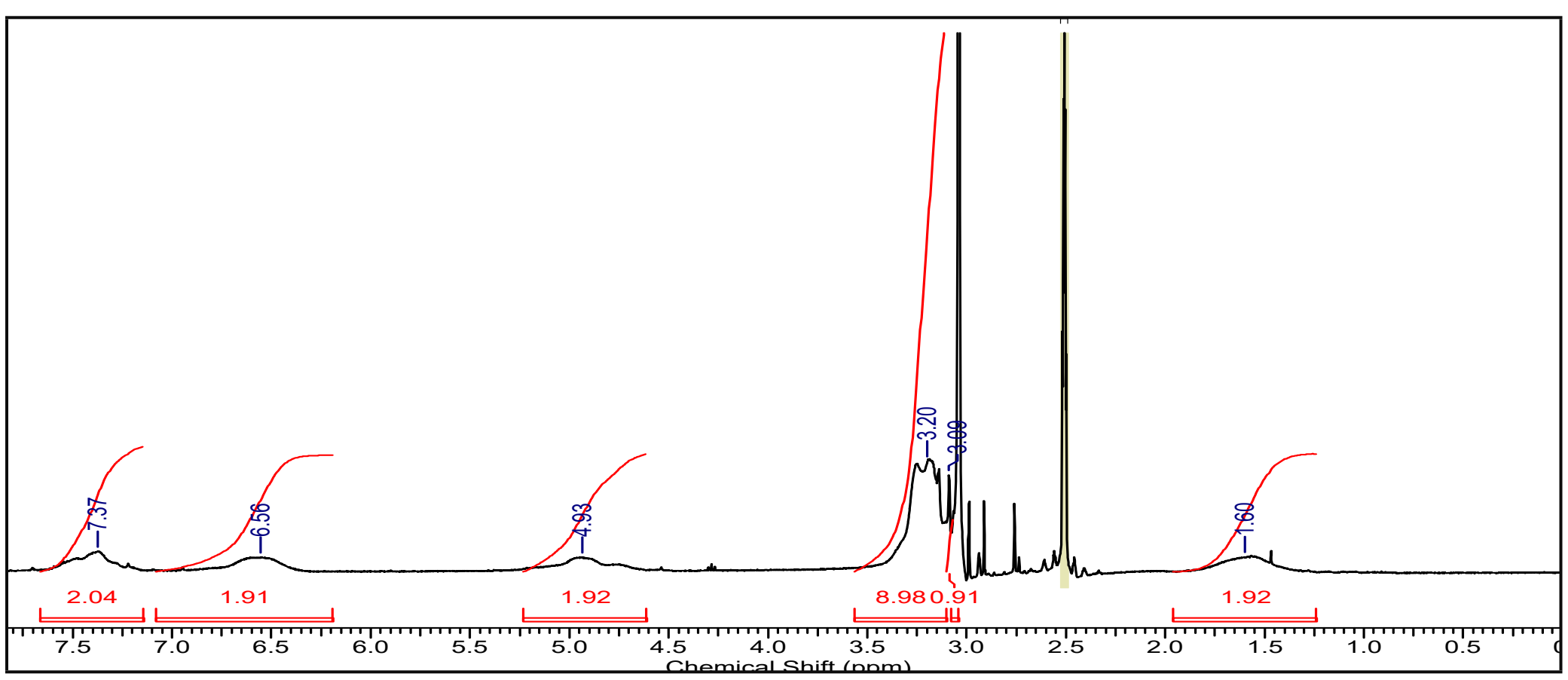

Figure S. 2 ^{1}HNMR for P[4-VBTMA][Cl]

P[4-VBTEA][Cl]; δ_H(600 MHz; DMSO): = 9.05 (-CH_2 -C *(CH)₂* Ar), 8.25 (-CH-C *(CH)₂* -CH Ar), 7.12 (N-*CH₂*CH(Ar), 3.09 (N-*CH₂*CH_3), 2.33 (C-*CH*-$(CH_2)_2$), 2.08 (N-*CH₂*CH_3), 1.20 (C-CH-*CH₂*). Elemental analysis: % actual C, 70.05; H, 10.58; N, 5.63 $(C_{15}H_{26}NCl)_n$. % theoretical C, 70.42; H, 10.34; N, 5.48. Mass fraction of water 223×10^{-6}.

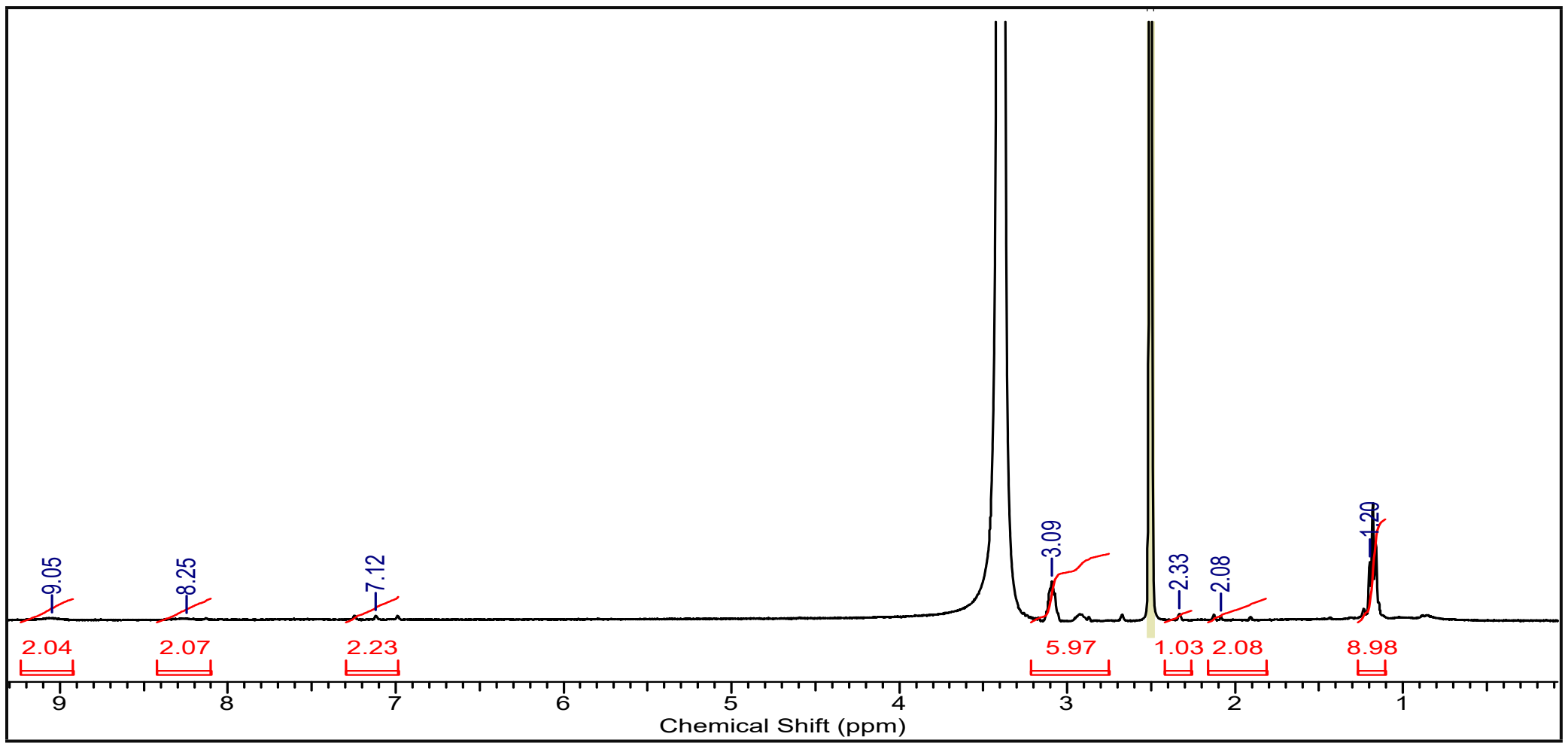

Figure S. 3 ^{1}HNMR for P[4-VBTEA][Cl]

P[4-MBP(DMA)₃][Cl]; δ_H(600 MHz; DMSO): = 7.99-8.64 (-CH_2 -C *(CH)₂* Ar), 7.51(CH-C *(CH)₂* Ar), 5.94 (N-*CH₂*-C), 4.82 (N-(*CH₃*)₂), 4.19 (P-N-*CH₃*)₂), 2.77 (C-CH-*CH₂*). Elemental analysis: % actual C, 56.49; H, 9.40; N, 13.11 $(C_{15}H_{29}N_3PCl)_n$. % theoretical C, 56.68; H, 9.20; N, 13.22. Mass fraction of water 241×10^{-6}.

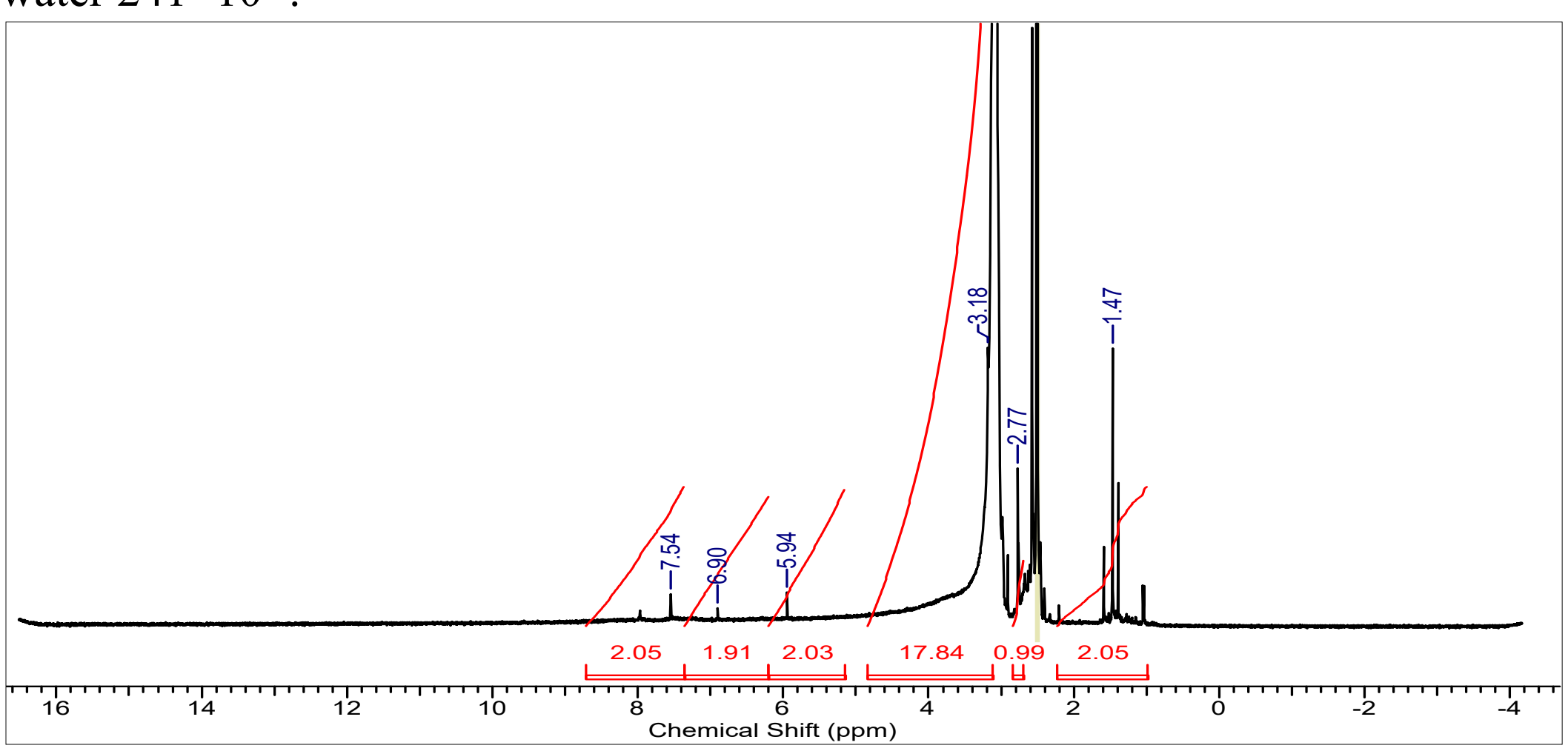

Figure S. 4 ^{1}HNMR for P[4-MBP(DMA)₃][Cl]

P[4-VBPDMA-TMA][Cl]; δ_H(600 MHz; DMSO): = 7.54 ($-CH_2-C$ *$(CH)_2$* Ar), 6.90(CH-C *$(CH)_2$* Ar), 5.94 (P-*CH_2*-C), 3.18 (C-*CH*-CH_2), 2.77 (P-N-*$CH_3)_2$*), 1.47 (C-CH-*CH_2*). Elemental analysis: % actual C, 56.45; H, 9.42; N, 13.09 $(C_{15}H_{29}N_3PCl)_n$. % theoretical C, 56.68; H, 9.20; N, 13.22. Mass fraction of water 229×10^{-6}.

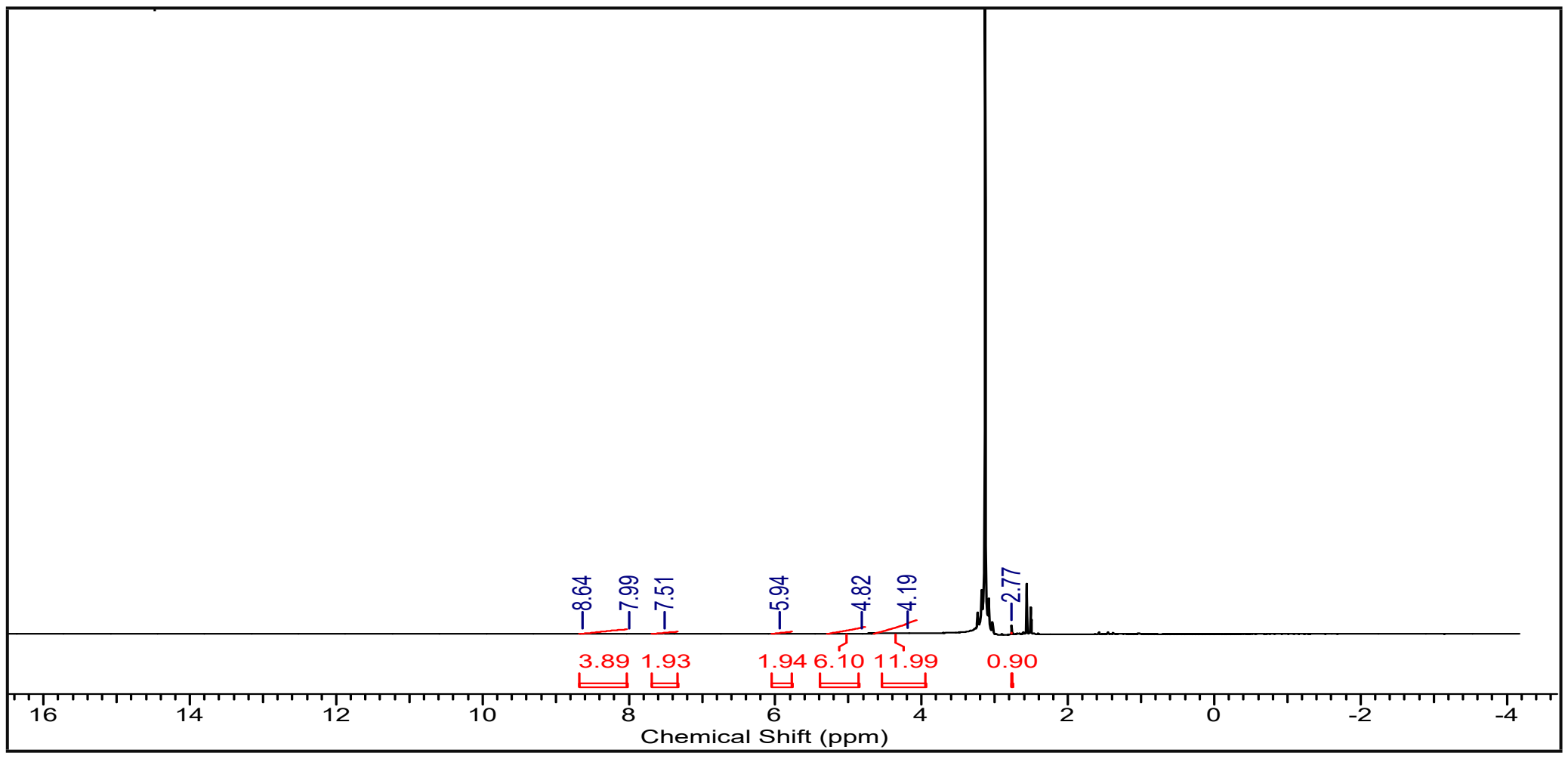

Figure S. 5 ^{1}HNMR for P[4-VBPDMA-TMA][Cl]

P[MOETMA][Cl]; δ_H(600 MHz; DMSO): = 4.44 (N-*CH_2*-CH_2), 3.92(N-CH_2-*CH_2*), 3.38-3.61 (N-*$(CH_3)_3$*), 3.28 (C-*CH*-CH_2), 0.86 (C-CH-*CH_2*). Elemental analysis: % actual C, 48.95; H, 9.61; N, 7.03 $(C_8H_{18}NO_2Cl)_n$. % theoretical C, C, 49.10; H, 9.27; N, 7.16. Mass fraction of water 235×10^{-6}.

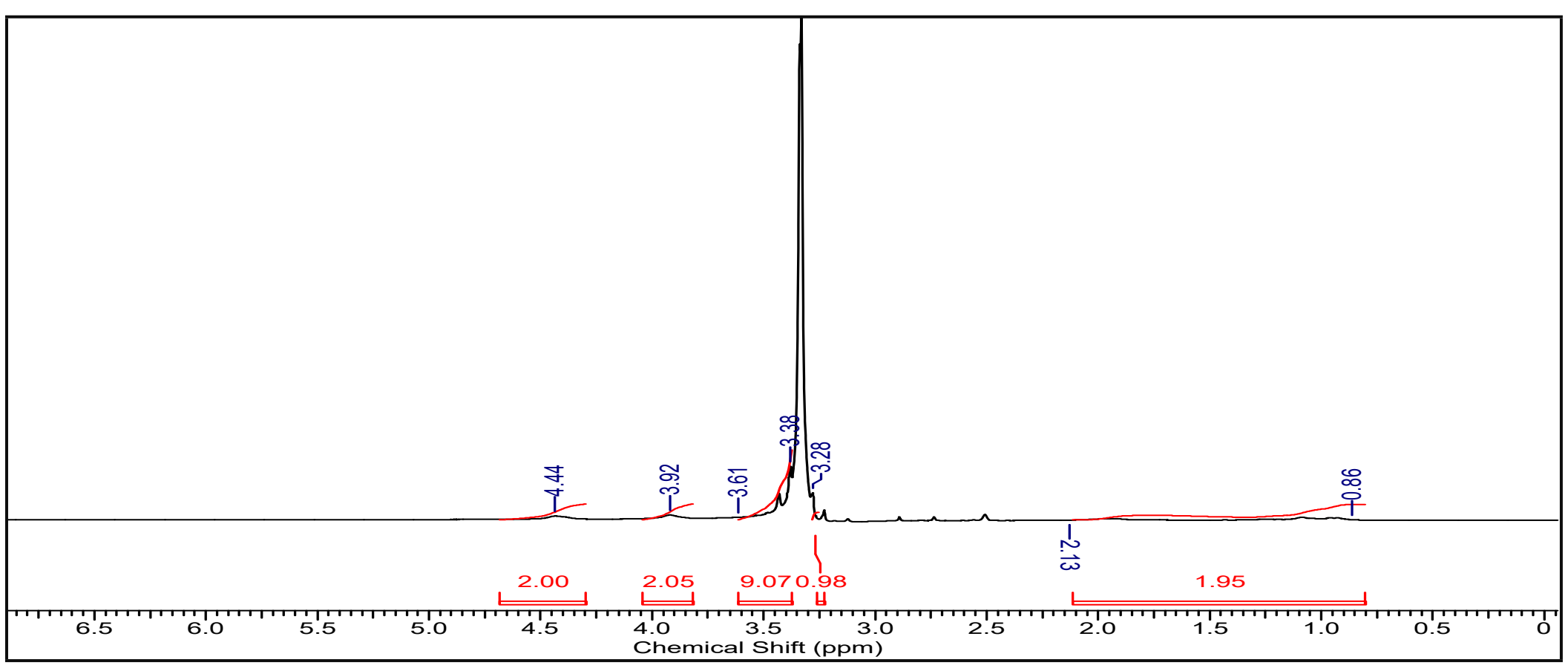

Figure S. 6 ^{1}HNMR for P[MOETMA][Cl]

P[AyPyr][Cl]; δ_H(600 MHz; DMSO): = 9.19 (N-*CH*-CH-CH), 8.43 (N-CH-CH-*CH*-), 8.31 (N-CH-*CH*-CH), 7.92 (H-C-CH-*C*-H), 2.49 (H-*CH*-C-H), 8.31 (N-CH-*CH*-CH), 2.19 (H-C-CH_2-H), 1.75 (H-CH_2-*CH*- H), 5.03 (N-CH_2-CH- *CH*). Elemental analysis: % actual C, 62.68; H, 8.41; N, 8.05 $(C_8H_{10}NCl)_n$. % theoretical C, 62.97; H, 8.22; N, 8.16. Mass fraction of water 265×10^{-6}.

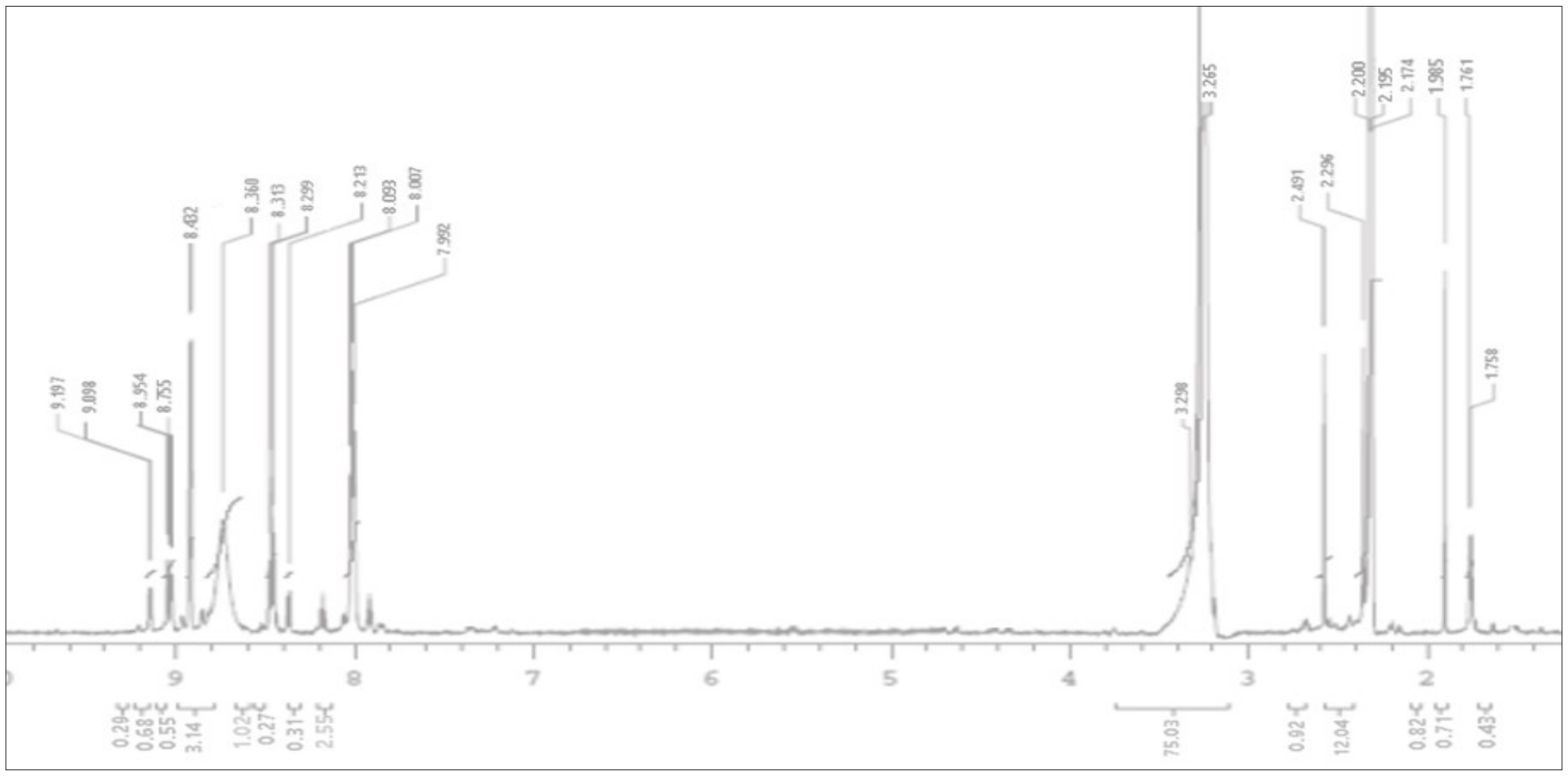

Figure S. 7 ^{1}HNMR spectra for poly P[AyPyr][Cl]

P[AyPrCNIm][Cl]; δ_H(600 MHz; DMSO): = 9.20 (N-*CH*-N), 7.78 (N-*CH*-CH-N), 7.61 (N-CH-*CH*-N), 4.78 (N-CH_2-*CH*- CH), 4.39 (N-CH_2-CH_2- CN), 3.4 (N-CH_2-CH-*CH*), 3.1 (N-CH_2-CH- CH), 2.3 (N-CH_2-CH_2- CN). Elemental analysis: % actual C, 56.01; H, 7.71; N, 19.049 $(C_9H_{12}N_3Cl)_n$. % theoretical C, 56.20; H, 7.55; N, 19.66. Mass fraction of water 238×10^{-6}.

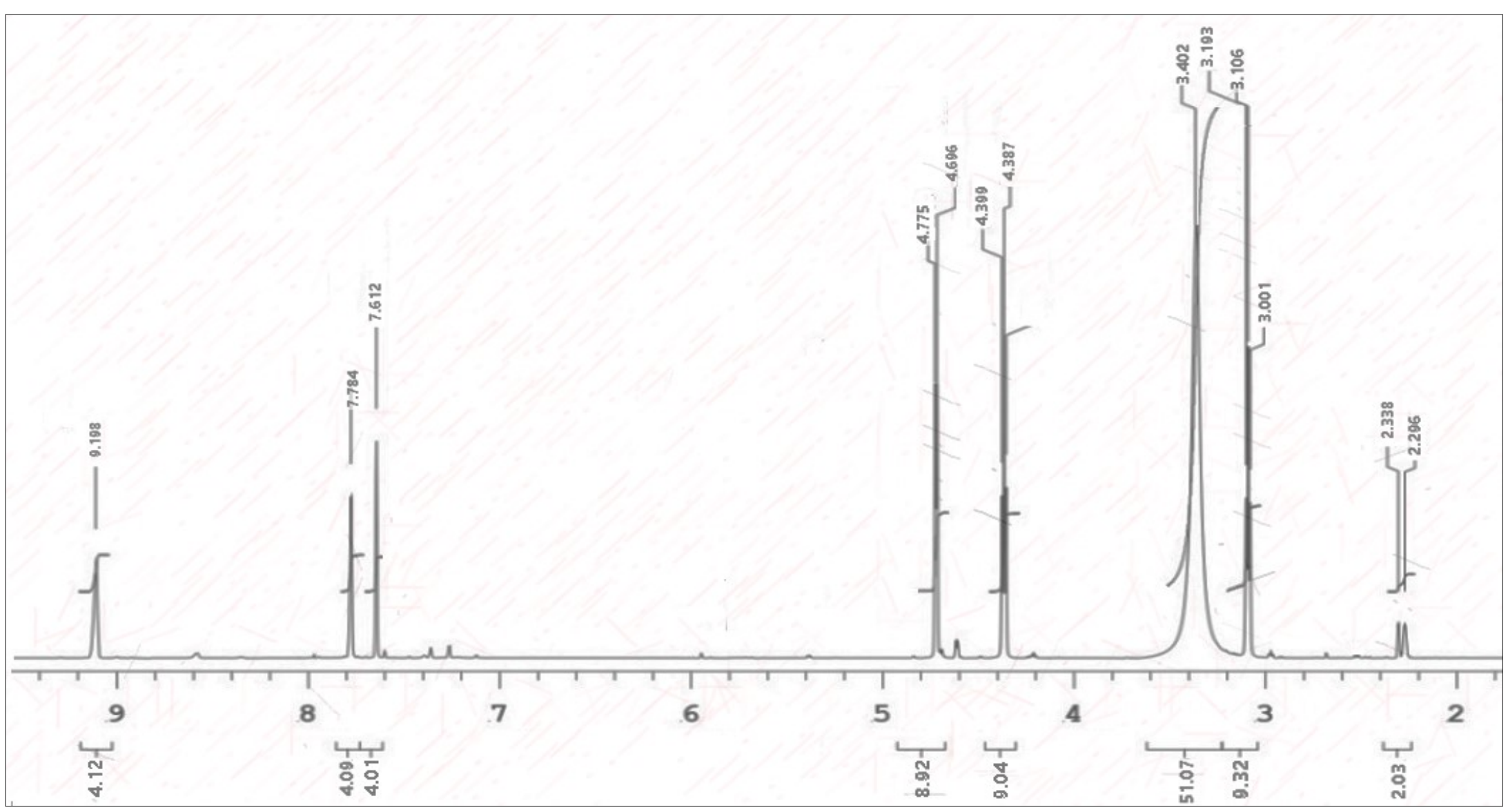

Figure S. 8 ^{1}H NMR spectra for P[AyPrCNIm][Cl]

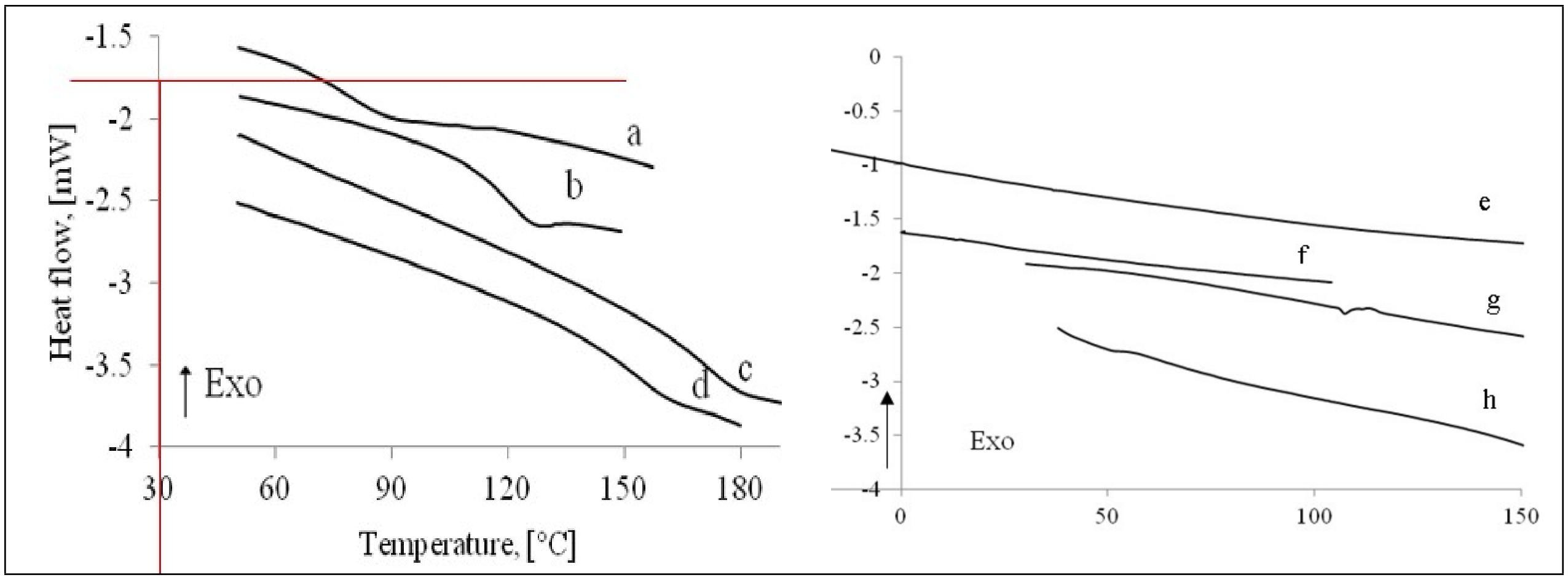

Figure S. 9 DSC curves for PILs: (a) P[MOETMA][Cl],] (b) P[AyPyr] [Cl], (c) P[4-VBTEA][Cl], (d) P[4-VBT MA][Cl], (e) P[AyTHEA][Cl], (f) P[4-MBP(DMA)$_3$][Cl], (g) P[4-VBPDMA-TMA][Cl] and (h) P[AyPrCNIm][Cl]

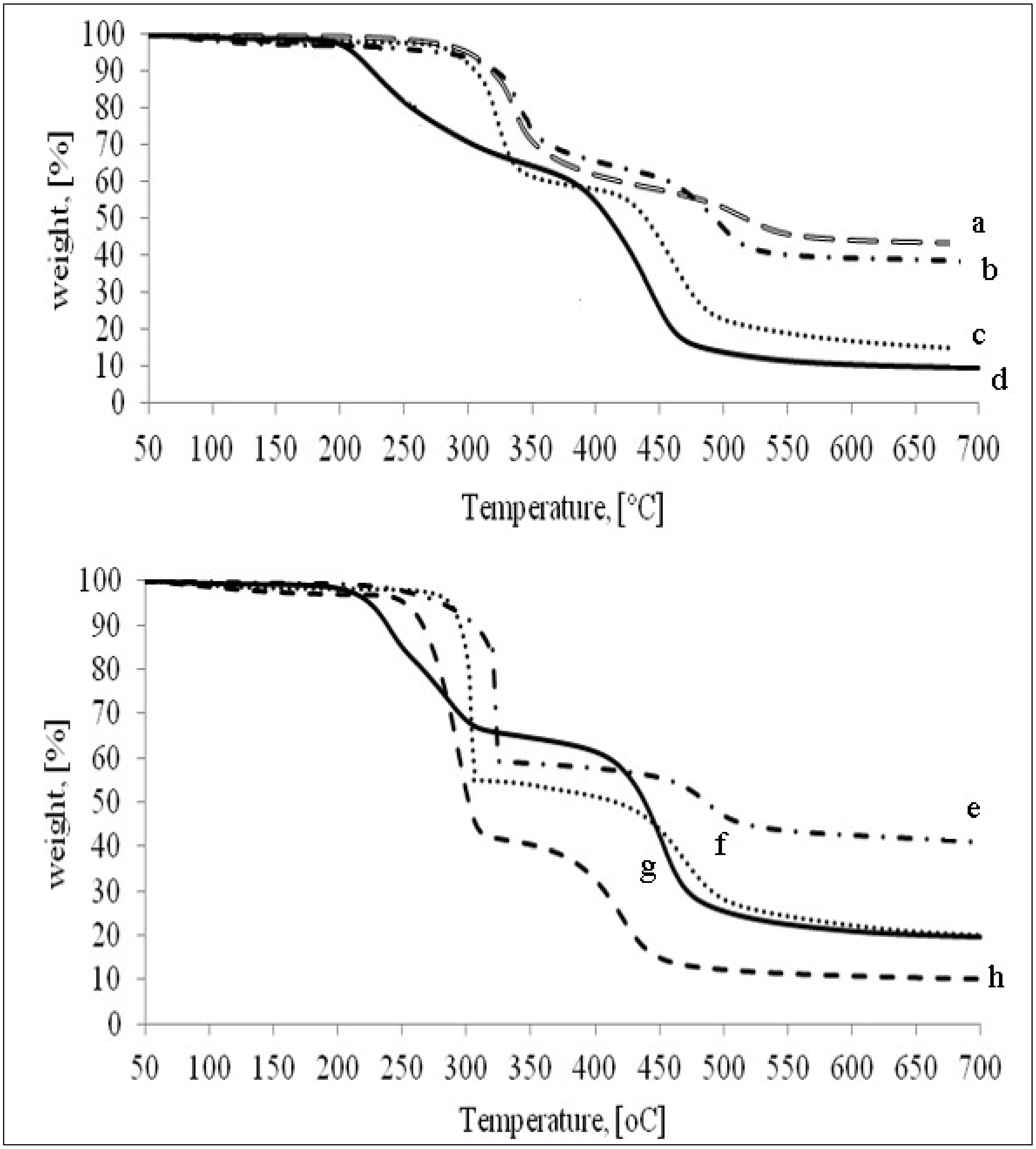

Figure S. 10 TGA graph for PILs: (a) P[MOETMA][Cl],] (b) P[AyPyr] [Cl], (c) P[4-VBTEA][Cl], (d) P[4-VBTMA][Cl], (e) P[AyTHEA][Cl], (f) P[4-MBP(DMA)$_3$][Cl], (g) P[4-VBPDMA-TMA][Cl], and (h) P[AyPrCNIm][Cl]

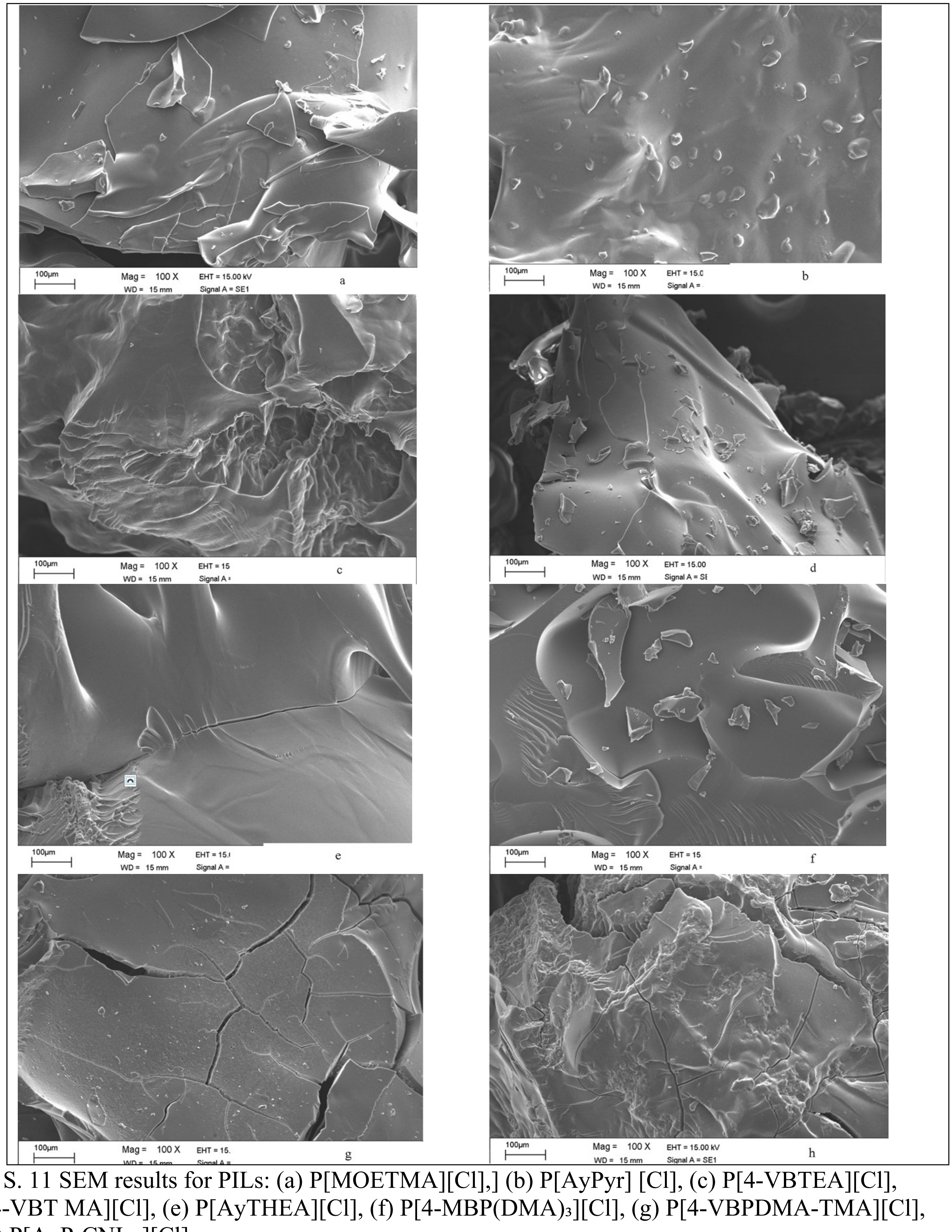

Figure S. 11 SEM results for PILs: (a) P[MOETMA][Cl],] (b) P[AyPyr] [Cl], (c) P[4-VBTEA][Cl], (d) P[4-VBT MA][Cl], (e) P[AyTHEA][Cl], (f) P[4-MBP(DMA)$_3$][Cl], (g) P[4-VBPDMA-TMA][Cl], and (h) P[AyPrCNIm][Cl]

Table S. 1 Experimental solubility data for CO_2 in P[AyTHEA][Cl] at different pressures

Time (min)	20 bars x_{CO_2}	Time (min)	15 bars x_{CO_2}	Time	10 bars x_{CO_2}	Time (min)	5 bars x_{CO_2}	Time	1 bar x_{CO_2}
0	0	0	0	0	0	0	0	0	0
8.6	0.0657	19.1	0.1272	11.2	0.0331	4.8	0.0104	45.0	0.0107
14.4	0.1234	46.4	0.2242	44.9	0.1360	10.6	0.0219	67.5	0.0137
28.8	0.2096	59.9	0.2522	59.0	0.1743	17.3	0.0384	90.5	0.0160
49.0	0.2905	77.0	0.2886	81.4	0.2212	23.0	0.0503	137.7	0.0184
60.5	0.3403	99.8	0.3314	106.7	0.2672	29.8	0.0627	188.5	0.0196
100.8	0.4166	119.7	0.3654	143.2	0.2990	48.5	0.0867	208.2	0.0202
149.8	0.4695	145.4	0.3950	176.9	0.3170	73.3	0.1069	225.1	0.0206
169.9	0.4848	182.4	0.4320	224.6	0.3308	81.4	0.1149	244.8	0.0211
227.5	0.5037	208.1	0.4508	275.2	0.3403	103.0	0.1298	275.8	0.0216
290.9	0.5215	228.0	0.4576	297.6	0.3454	126.0	0.1381	287.0	0.0218
322.6	0.5311	256.5	0.4669	321.3	0.3508	137.8	0.1434	323.6	0.0222
391.7	0.5449	290.7	0.4759	381.9	0.3614	199.5	0.1618	337.7	0.0225
455.0	0.5566	383.6	0.4860	426.8	0.3674	287.0	0.1745	368.6	0.0228
541.4	0.5654	458.2	0.4889	452.1	0.3710	346.1	0.1815	385.5	0.0229
601.9	0.5694	488.5	0.4895	491.4	0.3749	399.6	0.1864	410.8	0.0229
616.3	0.5689	529.9	0.4897	544.8	0.3830	526.2	0.1947	486.8	0.0234
648.0	0.5716	598.9	0.4906	572.8	0.3860	571.2	0.1961	542.7	0.0236
676.8	0.5730	637.6	0.4949	609.3	0.3895	593.8	0.1973	579.7	0.0236

Table S. 2 Experimental solubility data for CO_2 in P[4-VBTMA][Cl] at different pressures

Time (min)	20 bars x_{CO_2}	Time (min)	15 bars x_{CO_2}	Time (min)	10 bars x_{CO_2}	Time (min)	5 bars x_{CO_2}	Time (min)	1 bar x_{CO_2}
0	0	0	0	0	0	0	0	0	0
7.7	0.0349	17.9	0.0720	10.1	0.0193	7.5	0.0056	35.0	0.0048
12.8	0.0655	43.4	0.1269	40.3	0.0793	16.5	0.0117	51.8	0.0061
25.5	0.1112	53.6	0.1427	52.9	0.1016	27.0	0.0206	63.5	0.0071
43.4	0.1541	68.9	0.1633	73.1	0.1227	36.0	0.0269	98.6	0.0083
53.6	0.1806	89.3	0.1836	95.8	0.1403	52.1	0.0336	146.7	0.0090
89.3	0.2211	107.1	0.1983	128.5	0.1570	77.4	0.0464	177.6	0.0093
132.6	0.2491	130.1	0.2144	158.8	0.1664	99.8	0.0556	192.0	0.0096
150.5	0.2594	163.2	0.2253	201.6	0.1737	119.7	0.0615	208.8	0.0098
201.5	0.2673	186.2	0.2306	247.0	0.1787	162.4	0.0715	235.2	0.0100
257.6	0.2767	204.0	0.2341	267.1	0.1794	184.5	0.0756	244.8	0.0101
285.6	0.2818	229.5	0.2389	299.9	0.1842	201.7	0.0778	276.0	0.0103
346.8	0.2891	260.1	0.2434	342.7	0.1864	233.7	0.0815	288.0	0.0105
402.9	0.2954	354.5	0.2486	383.0	0.1909	250.9	0.0833	314.4	0.0107
479.4	0.3000	423.3	0.2501	405.7	0.1933	302.6	0.0866	328.8	0.0108
533.0	0.3021	451.4	0.2504	441.0	0.1953	349.3	0.0890	350.4	0.0110
545.7	0.3031	489.6	0.2505	488.9	0.1989	460.0	0.0922	415.2	0.0113
573.8	0.3058	553.4	0.2509	514.1	0.2005	499.4	0.0936	470.3	0.0115
599.3	0.3078	589.1	0.2532	546.8	0.2023	519.1	0.0938	494.4	0.0116

Table S. 3 Experimental solubility data for CO_2 in P[4-VBTEA][Cl] at different pressures

Time (min)	20 bars x_{CO_2}	Time (min)	15 bars x_{CO_2}	Time	10 bars x_{CO_2}	Time	5 bars x_{CO_2}	Time	1 bar x_{CO_2}
0	0	0	0	0	0	0	0	0	0
7.9	0.0295	18.7	0.0639	10.2	0.0147	7.5	0.0047	38.4	0.0044
13.2	0.0554	45.4	0.1126	40.8	0.0604	18.5	0.0098	57.6	0.0056
26.4	0.0941	56.1	0.1266	53.6	0.0774	31.3	0.0172	70.5	0.0066
44.9	0.1304	72.1	0.1449	74.0	0.0935	41.8	0.0226	108.0	0.0076
55.4	0.1528	93.5	0.1629	96.9	0.1069	53.9	0.0281	160.8	0.0081
92.4	0.1870	112.1	0.1760	130.1	0.1196	77.4	0.0389	177.6	0.0082
137.3	0.2108	136.2	0.1902	160.7	0.1268	96.7	0.0463	192.0	0.0083
155.8	0.2195	170.9	0.1999	204.0	0.1323	112.9	0.0516	208.8	0.0085
208.6	0.2262	194.9	0.2046	249.9	0.1361	138.6	0.0593	235.2	0.0086
266.6	0.2341	213.6	0.2077	270.3	0.1367	162.0	0.0631	244.8	0.0088
295.7	0.2385	240.3	0.2119	303.5	0.1403	182.0	0.0667	276.0	0.0090
359.0	0.2446	272.3	0.2160	346.8	0.1420	242.3	0.0731	288.0	0.0092
417.1	0.2499	371.1	0.2206	387.6	0.1454	260.1	0.0747	314.4	0.0094
496.3	0.2539	443.2	0.2219	410.6	0.1453	313.7	0.0777	328.8	0.0095
551.8	0.2556	472.6	0.2222	446.3	0.1468	362.1	0.0798	359.2	0.0097
565.0	0.2565	512.6	0.2222	494.7	0.1516	476.9	0.0839	415.2	0.0100
594.0	0.2587	579.4	0.2226	520.2	0.1528	517.7	0.0843	482.4	0.0103
620.4	0.2605	616.8	0.2246	553.4	0.1624	538.1	0.0854	494.4	0.0103

Table S. 4 Experimental solubility data for CO_2 in P[4-MBP(DMA)₃][Cl] at different pressures

Time (min)	20 bars x_{CO_2}	Time (min)	15 bars x_{CO_2}	Time (min)	10 bars x_{CO_2}	Time (min)	5 bars x_{CO_2}	Time (min)	1 bar x_{CO_2}
0	0	0	0	0	0	0	0	0	0
11.2	0.0616	29.4	0.1120	11.5	0.0295	33.5	0.0448	22.1	0.0043
33.5	0.1647	57.2	0.1799	49.0	0.1112	50.4	0.0596	42.8	0.0080
78.1	0.2585	75.6	0.2062	74.9	0.1407	76.8	0.0761	61.2	0.0098
93.0	0.2736	96.6	0.2289	115.2	0.1715	108.0	0.0883	81.2	0.0112
118.8	0.2997	134.4	0.2648	155.5	0.1907	129.6	0.0948	135.3	0.0129
155.1	0.3192	168.8	0.2877	207.4	0.2087	163.2	0.1003	152.5	0.0132
205.2	0.3414	194.0	0.2969	225.0	0.2123	206.4	0.1032	163.2	0.0135
221.8	0.3474	226.8	0.3058	279.3	0.2201	230.4	0.1044	180.0	0.0138
257.0	0.3537	247.0	0.3092	306.5	0.2246	256.8	0.1053	206.4	0.0143
279.7	0.3560	292.3	0.3116	351.0	0.2278	283.2	0.1068	216.0	0.0144
332.6	0.3634	322.6	0.3140	370.8	0.2301	324.0	0.1078	247.2	0.0149
365.4	0.3661	360.4	0.3161	425.2	0.2339	340.8	0.1087	259.2	0.0150
408.2	0.3689	405.7	0.3170	442.5	0.2356	393.6	0.1093	285.6	0.0153
458.6	0.3714	418.3	0.3175	489.5	0.2407	410.4	0.1108	300.0	0.0155
496.4	0.3746	453.6	0.3219	516.6	0.2434	451.2	0.1117	345.6	0.0160
539.3	0.3782	547.2	0.3218	541.4	0.2447	477.6	0.1120	386.4	0.0163
589.7	0.3781	577.1	0.3224	561.1	0.2448	494.4	0.1128	453.6	0.0166
619.9	0.3775	604.8	0.3226	573.5	0.2448	519.5	0.1136	501.6	0.0167
642.6	0.3775	625.0	0.3226	608.1	0.2448	573.6	0.1188	566.4	0.0168

Table S. 5 Experimental solubility data for CO_2 in P[4-VBPDMA-TMA][Cl] at different pressures

Time (min)	20 bars x_{CO_2}	Time (min)	15 bars x_{CO_2}	Time (min)	10 bars x_{CO_2}	Time (min)	5 bars x_{CO_2}	Time (min)	1 bar x_{CO_2}
0	0	0	0	0	0	0	0	0	0
7.2	0.0697	25.5	0.1570	9.6	0.0354	7.5	0.0148	38.4	0.0108
12.0	0.1310	40.8	0.2385	38.4	0.1457	16.5	0.0311	57.6	0.0138
24.0	0.2225	50.4	0.2683	50.4	0.2005	27.0	0.0545	71.3	0.0161
40.8	0.3083	64.8	0.3070	69.6	0.2421	36.0	0.0713	108.0	0.0187
50.4	0.3611	84.0	0.3452	91.2	0.2958	44.6	0.0888	160.8	0.0207
84.0	0.4421	100.8	0.3728	122.4	0.3311	69.7	0.1228	177.6	0.0212
124.8	0.4983	122.4	0.4030	151.2	0.3510	109.2	0.1515	192.0	0.0215
141.6	0.5148	153.6	0.4405	192.0	0.3663	147.1	0.1628	208.8	0.0220
189.6	0.5428	175.2	0.4595	235.2	0.3768	170.3	0.1840	229.3	0.0224
242.4	0.5598	192.0	0.4700	254.4	0.3783	193.5	0.1957	244.8	0.0227
268.8	0.5637	216.0	0.4849	285.6	0.3884	211.6	0.2001	276.0	0.0230
326.4	0.5782	244.8	0.4942	326.4	0.3931	245.1	0.2157	288.0	0.0231
379.2	0.5907	333.6	0.5047	364.8	0.4025	293.8	0.2205	314.4	0.0233
451.2	0.6001	398.4	0.5134	386.4	0.4060	361.6	0.2293	328.8	0.0235
501.6	0.6042	424.8	0.5159	420.0	0.4130	426.0	0.2355	376.7	0.0238
513.6	0.6062	460.8	0.5179	465.6	0.4195	482.5	0.2477	415.2	0.0241
540.0	0.6116	520.8	0.5189	489.6	0.4229	523.7	0.2477	458.3	0.0243
564.0	0.6156	554.4	0.5234	520.8	0.4267	544.4	0.2519	494.4	0.0244

Table S. 6 Experimental solubility data for CO_2 in P[MOETMA][Cl] at different pressures

Time (min)	20 bars x_{CO_2}	Time (min)	15 bars x_{CO_2}	Time (min)	10 bars x_{CO_2}	Time (min)	5 bars x_{CO_2}	Time (min)	1 bar x_{CO_2}
0	0	0	0	0	0	0	0	0	0
7.7	0.0464	21.8	0.1012	12.0	0.0258	5.4	0.0078	38.4	0.0083
12.8	0.0872	47.9	0.1784	41.3	0.1060	11.9	0.0164	57.6	0.0106
25.5	0.1480	58.0	0.2007	54.2	0.1359	19.4	0.0287	78.3	0.0120
43.4	0.2051	71.3	0.2296	69.6	0.1641	25.9	0.0376	108.0	0.0133
53.6	0.2403	88.6	0.2582	91.2	0.1875	33.5	0.0469	160.8	0.0147
89.3	0.2942	106.3	0.2789	128.5	0.2098	46.4	0.0648	177.6	0.0151
132.6	0.3316	129.1	0.3015	158.8	0.2225	62.4	0.0800	192.0	0.0155
150.5	0.3452	162.0	0.3168	201.6	0.2322	71.8	0.0859	208.8	0.0156
201.5	0.3557	184.8	0.3242	247.0	0.2388	99.0	0.0971	235.2	0.0161
257.6	0.3682	202.6	0.3291	267.1	0.2398	121.5	0.1033	244.8	0.0163
285.6	0.3751	227.9	0.3358	299.9	0.2453	142.7	0.1067	276.0	0.0167
346.8	0.3847	258.3	0.3423	342.7	0.2492	171.0	0.1108	288.0	0.0169
402.9	0.3931	351.9	0.3496	383.0	0.2549	244.8	0.1164	314.4	0.0171
479.4	0.3993	420.3	0.3517	405.7	0.2562	295.2	0.1210	328.8	0.0174
533.0	0.4020	448.2	0.3521	441.0	0.2589	340.8	0.1243	376.7	0.0177
545.7	0.4033	486.1	0.3522	488.9	0.2659	403.9	0.1273	425.6	0.0179
573.8	0.4069	549.4	0.3536	514.1	0.2680	475.0	0.1307	470.3	0.0181
599.3	0.4096	584.9	0.3559	546.8	0.2705	506.4	0.1329	494.4	0.0182

Table S. 7 Experimental solubility data for CO_2 in P[AyPyr][Cl] at different pressures

Time (min)	20 bars x_{CO_2}	Time (min)	15 bars x_{CO_2}	Time (min)	10 bars x_{CO_2}	Time (min)	5 bars x_{CO_2}	Time (min)	1 bar x_{CO_2}
0	0	0	0	0	0	0	0	0	0
7.7	0.0241	17.9	0.0520	10.1	0.0121	12.3	0.0038	38.4	0.0037
12.8	0.0453	43.4	0.0916	40.3	0.0496	27.1	0.0085	57.6	0.0047
25.5	0.0770	53.6	0.1030	52.9	0.0636	44.3	0.0148	73.1	0.0055
43.4	0.1067	68.9	0.1179	73.1	0.0768	59.0	0.0194	108.0	0.0063
53.6	0.1250	89.3	0.1326	95.8	0.0916	76.3	0.0242	160.8	0.0067
89.3	0.1530	107.1	0.1432	128.5	0.1036	105.8	0.0334	177.6	0.0069
132.6	0.1725	130.1	0.1548	158.8	0.1110	127.9	0.0413	192.0	0.0071
150.5	0.1796	163.2	0.1626	201.6	0.1158	140.2	0.0443	208.8	0.0071
201.5	0.1850	186.2	0.1664	247.0	0.1191	162.4	0.0501	235.2	0.0072
257.6	0.1916	204.0	0.1690	267.1	0.1196	184.5	0.0533	250.9	0.0073
285.6	0.1951	229.5	0.1724	299.9	0.1228	201.7	0.0545	276.0	0.0075
346.8	0.2002	260.1	0.1757	342.7	0.1243	233.7	0.0587	288.0	0.0077
402.9	0.2045	354.5	0.1795	383.0	0.1272	250.9	0.0600	314.4	0.0078
479.4	0.2077	423.3	0.1805	405.7	0.1271	302.6	0.0624	328.8	0.0079
533.0	0.2092	451.4	0.1807	441.0	0.1285	349.3	0.0641	350.4	0.0080
545.7	0.2098	489.6	0.1808	488.9	0.1326	460.0	0.0674	415.2	0.0084
573.8	0.2117	553.4	0.1811	514.1	0.1337	499.4	0.0674	458.3	0.0085
599.3	0.2131	589.1	0.1827	546.8	0.1390	519.1	0.0686	494.4	0.0085

Table S. 8 Experimental solubility data for CO_2 in P[AyPrCNIm][[Cl] at different pressures

Time (min)	20 bars x_{CO_2}	Time (min)	15 bars x_{CO_2}	Time (min)	10 bars x_{CO_2}	Time (min)	5 bars x_{CO_2}	Time (min)	1 bar x_{CO_2}
0	0	0	0	0	0	0	0	0	0
12.6	0.0862	33.0	0.1606	13.9	0.0425	35.1	0.0626	21.6	0.0056
37.8	0.2305	75.4	0.2580	59.2	0.1602	56.7	0.0834	50.0	0.0104
88.2	0.3619	99.0	0.2956	90.5	0.2028	86.4	0.1065	70.6	0.0128
97.5	0.3830	126.5	0.3372	139.2	0.2596	121.5	0.1235	97.0	0.0147
128.7	0.4196	150.4	0.3594	187.9	0.2886	145.8	0.1326	132.0	0.0168
155.1	0.4468	188.9	0.3968	250.6	0.3038	183.6	0.1422	148.8	0.0172
200.6	0.4779	217.1	0.4140	316.7	0.3151	232.2	0.1515	163.2	0.0177
232.3	0.4864	253.8	0.4309	393.2	0.3281	259.2	0.1570	180.0	0.0180
269.3	0.4951	276.4	0.4358	431.5	0.3368	272.9	0.1584	206.4	0.0186
293.0	0.4984	309.7	0.4391	477.1	0.3432	300.9	0.1606	216.0	0.0188
348.5	0.5088	341.8	0.4425	486.0	0.3451	344.3	0.1644	247.2	0.0194
382.8	0.5126	381.8	0.4454	557.3	0.3508	362.1	0.1657	259.2	0.0196
427.7	0.5165	429.9	0.4468	569.2	0.3519	418.2	0.1697	285.6	0.0200
480.5	0.5199	443.2	0.4474	582.1	0.3535	436.1	0.1704	300.0	0.0202
520.1	0.5245	504.6	0.4537	595.7	0.3574	479.4	0.1757	345.6	0.0208
565.0	0.5295	608.8	0.4536	604.4	0.3563	507.5	0.1778	386.4	0.0211
617.8	0.5294	611.4	0.4543	619.7	0.3563	525.3	0.1791	453.6	0.0215
649.4	0.5285	640.8	0.4546	625.0	0.3563	575.6	0.1826	501.6	0.0218

Table S. 9 First-order saturation model $x_{CO_2}(t)$ parameter for CO_2 absorption in synthesized PILs at various pressures.

PIL	P (bar)				
	20	15	10	5	1
P[AyTHEA][Cl]	0.5524	0.4901	0.3748	0.1897	0.0228
P[4-VBTMA][Cl]	0.2940	0.2485	0.1933	0.0940	0.0108
P[4-VBTEA][Cl]	0.2488	0.2204	0.1479	0.0859	0.0095
P[4-MBP(DMA)$_3$][Cl]	0.3695	0.3200	0.2389	0.1110	0.0159
P[4-VBPDMA-TMA][Cl]	0.5891	0.5137	0.4106	0.2454	0.0236
P[MOETMA][Cl]	0.3913	0.3514	0.2578	0.1263	0.0173
P[AyPyr][Cl]	0.2036	0.1793	0.1298	0.0723	0.0079
P[AyPrCNIm][Cl]	0.5181	0.4532	0.3502	0.1732	0.0208

Table S. 10 First-order saturation model rate constant parameter (k (min^{-1})) for CO_2 absorption in synthesized PILs at various pressures.

PIL	P (bar)				
	20	15	10	5	1
P[AyTHEA][Cl]	0.0146	0.0119	0.0105	0.0113	0.0125
P[4-VBTMA][Cl]	0.0164	0.0156	0.0130	0.0088	0.0146
P[4-VBTEA][Cl]	0.0158	0.0149	0.0127	0.0079	0.0142
P[4-MBP(DMA)$_3$][Cl]	0.0148	0.0137	0.0110	0.0147	0.0135
P[4-VBPDMA-TMA][Cl]	0.0174	0.0136	0.0126	0.0086	0.0148
P[MOETMA][Cl]	0.0164	0.0148	0.0132	0.0144	0.0143
P[AyPyr][Cl]	0.0164	0.0156	0.0121	0.0065	0.0143
P[AyPrCNIm][Cl]	0.0136	0.0111	0.0093	0.0104	0.0124

Table S. 11 First-order saturation model half-saturation time ($t_{½}$) parameter for CO_2 absorption in synthesized PILs at various pressures.

PIL	P (bar)				
	20	15	10	5	1
P[AyTHEA][Cl]	47.6	58.1	65.8	61.6	55.6
P[4-VBTMA][Cl]	42.4	44.5	53.3	78.7	47.4
P[4-VBTEA][Cl]	43.8	46.5	54.5	87.8	48.9
P[4-MBP(DMA)$_3$][Cl]	47.0	50.7	63.2	47.1	51.3
P[4-VBPDMA-TMA][Cl]	40.0	51.0	55.0	80.9	46.9
P[MOETMA][Cl]	42.4	47.0	52.6	48.1	48.5
P[AyPyr][Cl]	42.4	44.4	57.5	107.0	48.5
P[AyPrCNIm][Cl]	51.1	62.4	74.4	67.0	55.7

Table S. 12 Coefficient of determination (R^2) for the First-order saturation model parameters for CO_2 absorption in synthesized PILs at various pressures.

PIL	P (bar)				
	20	15	10	5	1
P[AyTHEA][Cl]	0.9918	0.9964	0.9942	0.9935	0.9886
P[4-VBTMA][Cl]	0.9912	0.9968	0.9932	0.9996	0.9729
P[4-VBTEA][Cl]	0.9911	0.9968	0.9886	0.9985	0.9642
P[4-MBP(DMA)$_3$][Cl]	0.9942	0.9987	0.9934	0.9926	0.9801
P[4-VBPDMA-TMA][Cl]	0.9920	0.9965	0.9938	0.9942	0.9931
P[MOETMA][Cl]	0.9912	0.9993	0.9914	0.9935	0.9789
P[AyPyr][Cl]	0.9912	0.9968	0.9929	0.9901	0.9691
P[AyPrCNIm][Cl]	0.9966	0.9975	0.9947	0.9876	0.9913

CO_2 Solubility measurement using MSB

To achieve precise weight measurements, all possible environmental disturbances affecting the sample were minimized and identified. The buoyancy phenomenon cannot be diminished or eliminated; thus, it was measured and adjusted. Furthermore, the atmospheric density enveloping the sample was assessed to evaluate the buoyancy impact; the gas phase density under the specified conditions (temperature, pressure) was computed using the Peng-Robinson equation of state (PR-EOS). Helium has been used as a measuring gas for volume assessment due to its superior qualities compared to other gasses. The solubility assessment was conducted in four primary sequential stages: blank measurement, drying, buoyancy, and solubility evaluations. The rationale and objectives of these measures are succinctly outlined here.

Blank measurement

The blank measurement was performed only once (for the first sample) to measure the absorption isotherm without sample in the MSB and the data obtained were used to determine the mass and volume of the empty sample container. Starting from vacuum the pressure was increased stepwise with nitrogen (also helium can be used). The documented information during the baseline measurement included the balance reading (m_{BAL}), the temperature (T), and the pressure (P) of the gas. The temperature and pressure measurements were used to determine the gas density using the Peng-Robinson equation of state (PR-EOS). The weigh of the empty sample container decreases with increasing pressure of the gas due to the buoyancy effect acting on the sample container. The buoyancy phenomenon (B) influences any object situated in a gaseous environment with density ρ. It is directly related to the density and is defined as the multiplication of the density and the volume (V) of the object as seen in equation1.

$$B = \rho \cdot V \qquad 1$$

The balance reading was graphed in relation to the gas density, ρ, (Fig S.9) in order to determine the mass and volume of the empty sample container, ($m_{SC}$and V_{SC}), (shown by the negative slope).

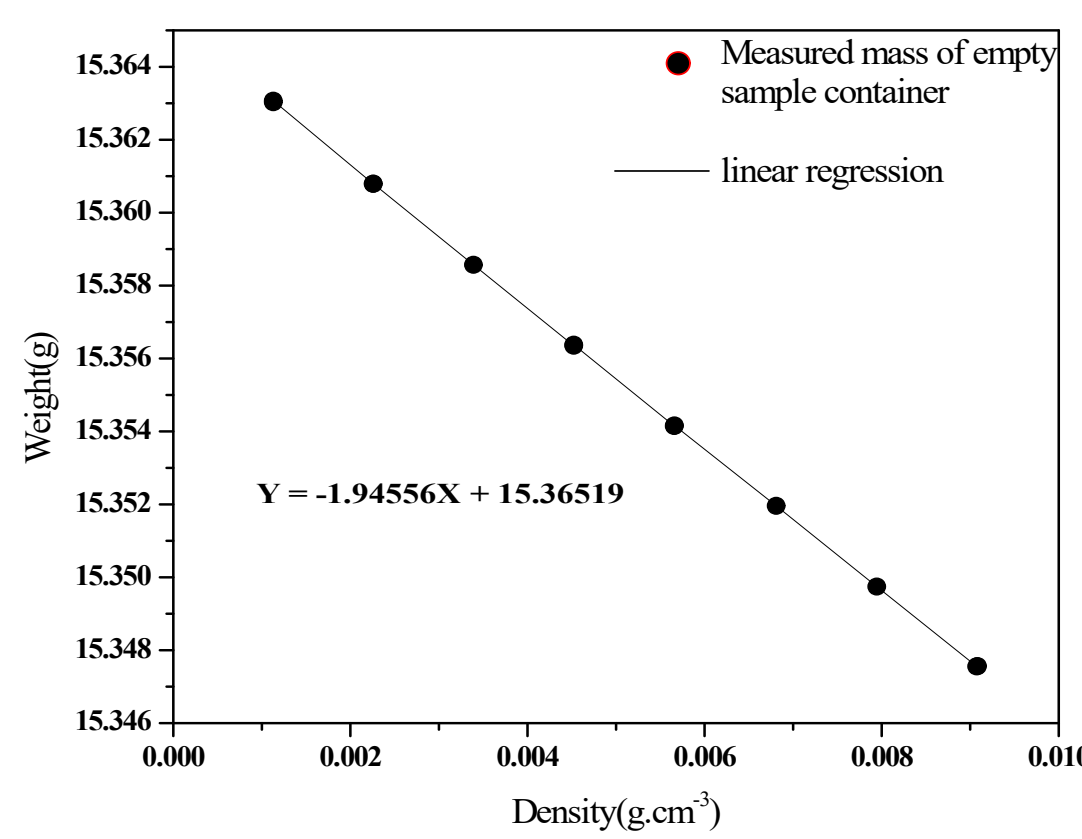

Figure S. 12 Example for result of the blank measurement

The linear regression of the measured masses can be written as:

$$m_{BAL} = m_{SC} - \rho \cdot V_{SC} \qquad 2$$

The values of the parameters of this linear function are the weight and volume of the empty sample container.

Drying

Each PIL was desiccated prior to the measurement of CO_2 solubility to guaranty optimal elimination of moisture and volatile substances. The sample was allocated over the three disks of the sample vessel (0.8-1.0 g) inside the MSB, and the measurement cell was sealed. A leak test was conducted by introducing helium to a designated pressure, after which the pressure controller was deactivated and the pressure gage was observed for a period to confirm the absence of gas leakage. The specimen was incrementally heated to 100 °C in a vacuum, and the mass was recorded by the MSB until no more weight loss was seen (Fig S.13), at which point the drying process was deemed finished. Subsequently, the sample was reduced to the measurement temperature. Upon completion of the drying process, this mass was quantified in a vacuum.

$$m_{SC+S} = m_{BAL}\ (P = 0, \rho = 0) \qquad 3$$

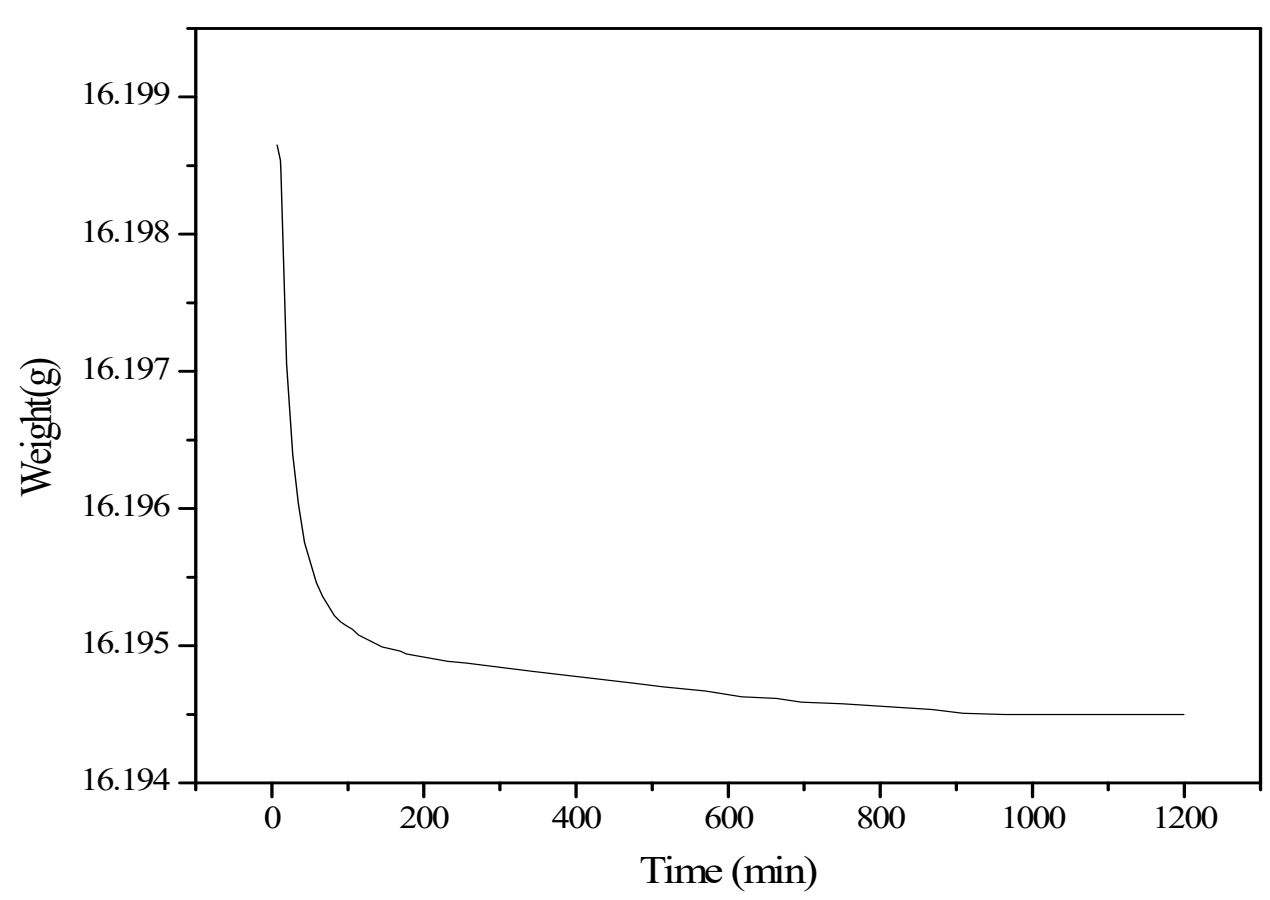

Figure S. 13 Example for the results of the drying process at 100 °C

Buoyancy measurement

Typically, the precise volume of the sample was not determined. To ascertain the volume of sample inside the MSB's sample container, a buoyancy measurement was conducted for each sample. The buoyancy measurement was conducted to adjust the sample weight's mass with the MSB during the solubility measurement, owing to the buoyancy influence exerted on it in the gaseous phase (equation 1).

The measurement was conducted by a gradual increase of pressure at a stable temperature using helium gas (helium exhibits little absorption and does not alter the sample's mass). The Peng-Robinson equation of state (PR-EOS) was used to determine the gas density at designated temperatures and pressures. Subsequently, the balance

measurement (m_{BAL}) was graphed against the gas density, resulting in a straight line with a negative slope (Fig S.14).

The decreasing trend in the recorded weights of the sample container (filled with sample) as gas pressure rises was attributed to the buoyant force exerted on both the sample container and the sample. The linear regression analysis of the recorded masses was conducted (Fig S.14).

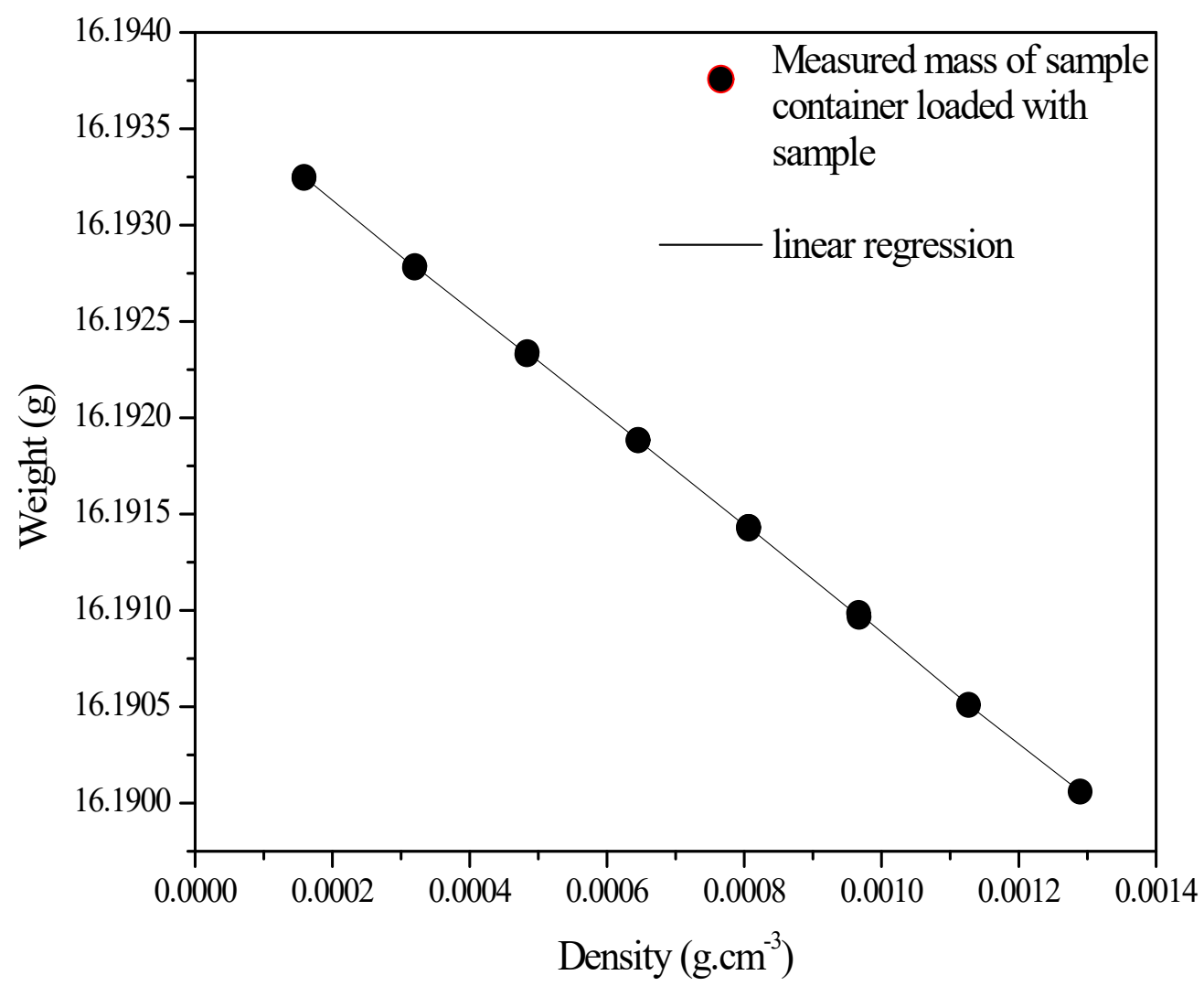

Figure S. 14 Example for the results of the buoyancy measurement

The linear function represents the mass of the sample container together with the sample in a vacuum ($m_{SC} + S$) subtracting the buoyancy effect. The buoyancy phenomenon arises from the combined volume of the empty sample container and the sample itself ($V_{SC} + S$). This gas is ascertained by deducting the volume of the vacant sample container (V_{SC}) established from the blank measurement). The sample's volume was subsequently determined:

$$m_{BAL} = m_{SC+S} - \rho \cdot V_{SC+S} \quad 4$$

The regression is shown in Figure S.14. The mass and volume of the sample were ascertained by the execution of buoyancy measurements.

Solubility measurement

Prior to conducting the solubility measurement and subsequent to the completion of the buoyancy evaluation, the MSB was evacuated. The solubility measurement was initiated at a steady temperature, with the gas pressure in the cell being gradually raised until the desired pressure was attained. The procedure was thereafter observed continually until the MSB indicated no more mass increase (or minimal increases) or the solubility measurement exhibited no further enhancement (Fig S.15), at which point the solubility measurement was deemed complete.

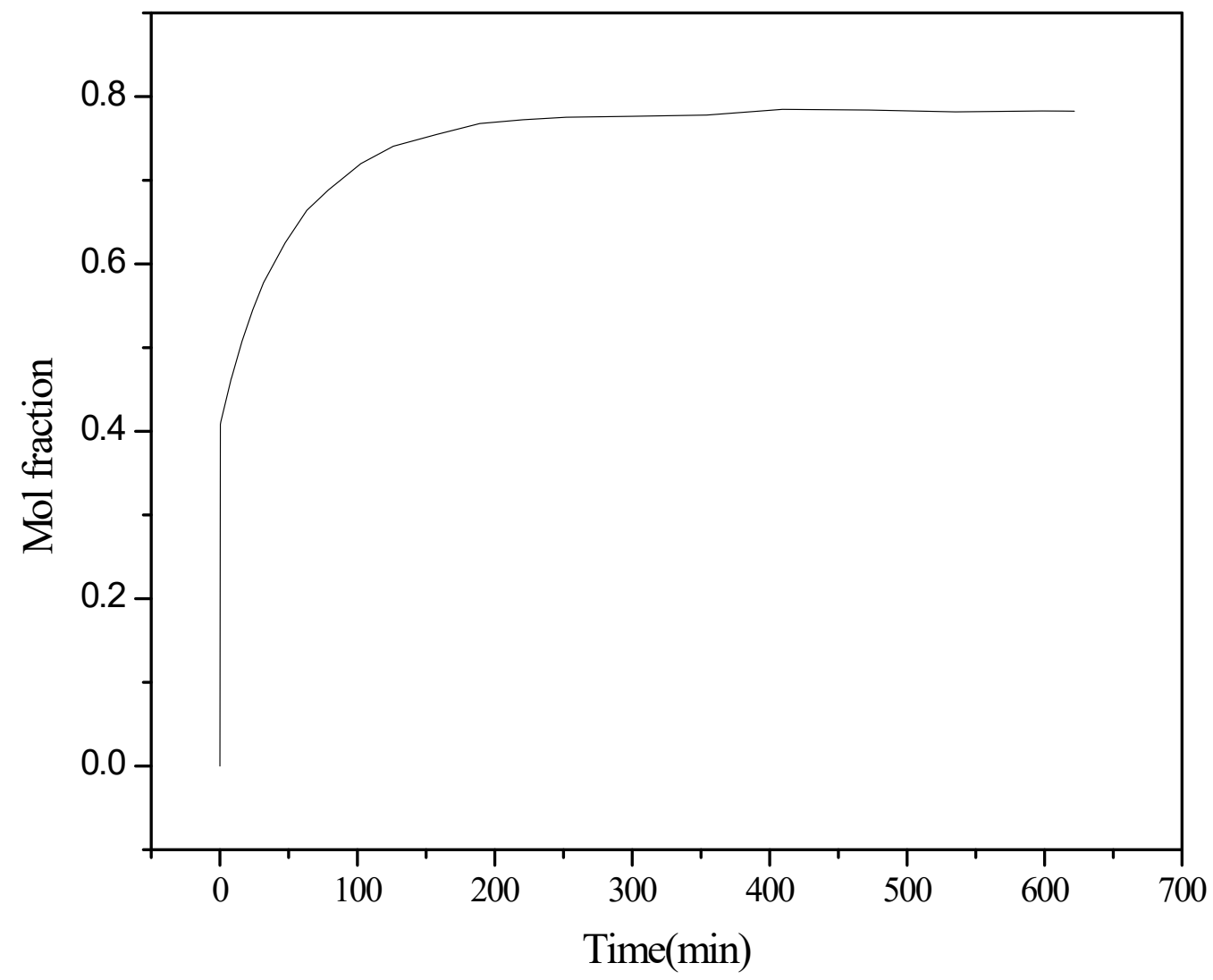

Figure S. 15 Example for the results of the solubility measurement

This data obtained from the solubility determination included the balance measurement (m_{BAL}), the temperature (T), and the gas pressure (P). The gas density was determined using the PR-EOS from this data. The documented balancing measurement (m_{BAL}) in the solubility assessment was adjusted for the buoyancy influence on the sample and its container. The adjustment was made by including the gas density (ρ, previously computed) and the sample container volume ($V_{SC} + S$), established during the prior buoyancy test) into the balance reading, as shown in equation 5.

$$m_{BAL.CORR} = m_{BAL} + \rho \cdot V_{SC+S} \quad 5$$

The buoyancy-adjusted mass refers to the combined mass of the sample and its container, whereas the mass of the sample itself is derived by deducting the mass of the empty container from this

$$m_S = m_{BAL.CORR} - m_{SC} \quad 6$$

The weight of the sample containing CO_2 was determined. The quantity of CO_2 absorbed by the specimen was subsequently determined in the following manner:

$$m_{CO_2} = m_{S+SC} - m_S \quad 7$$
